# Comparative transcriptome analysis of embryonic and adult stem cells with extended and limited differentiation capacity

**DOI:** 10.1186/gb-2007-8-8-r163

**Published:** 2007-08-06

**Authors:** Fernando Ulloa-Montoya, Benjamin L Kidder, Karen A Pauwelyn, Lucas G Chase, Aernout Luttun, Annelies Crabbe, Martine Geraerts, Alexei A Sharov, Yulan Piao, Minoru SH Ko, Wei-Shou Hu, Catherine M Verfaillie

**Affiliations:** 1 Stem Cell Institute, University of Minnesota, Minneapolis, MN 55455 USA; 2 Department of Chemical Engineering and Materials Science, University of Minnesota, Minneapolis, MN 55455 USA; 3 Stamcel Instituut, Katholieke Universiteit Leuven, Leuven, 3000 Belgium; 4 Developmental Genomics and Aging Section, Laboratory of Genetics, National Institute on Aging, NIH, Baltimore, MD 21224 USA

**Keywords:** Additional Data File, Inner Cell Mass, Definitive Endoderm, Mouse ESCs, Primitive Endoderm

## Abstract

**Background:**

Recently, several populations of postnatal stem cells, such as multipotent adult progenitor cells (MAPCs), have been described that have broader differentiation ability than classical adult stem cells. Here we compare the transcriptome of pluripotent embryonic stem cells (ESCs), MAPCs, and lineage-restricted mesenchymal stem cells (MSCs) to determine their relationship.

**Results:**

Applying principal component analysis, non-negative matrix factorization and k-means clustering algorithms to the gene-expression data, we identified a unique gene-expression profile for MAPCs. Apart from the ESC-specific transcription factor Oct4 and other ESC transcripts, some of them associated with maintaining ESC pluripotency, MAPCs also express transcripts characteristic of early endoderm and mesoderm. MAPCs do not, however, express Nanog or Sox2, two other key transcription factors involved in maintaining ESC properties. This unique molecular signature was seen irrespective of the microarray platform used and was very similar for both mouse and rat MAPCs. As MSC-like cells isolated under MAPC conditions are virtually identical to MSCs, and MSCs cultured in MAPC conditions do not upregulate MAPC-expressed transcripts, the MAPC signature is cell-type specific and not merely the result of differing culture conditions.

**Conclusion:**

Multivariate analysis techniques clustered stem cells on the basis of their expressed gene profile, and the genes determining this clustering reflected the stem cells' differentiation potential *in vitro*. This comparative transcriptome analysis should significantly aid the isolation and culture of MAPCs and MAPC-like cells, and form the basis for studies to gain insights into genes that confer on these cells their greater developmental potency.

**Electronic supplementary material:**

The online version of this article (doi:10.1186/gb-2007-8-8-r163) contains supplementary material, which is available to authorized users.

## Background

Multiple types of adult stem cell exist, of which the hematopoietic stem cell (HSC), which gives rise to cells of all hematopoietic lineages for the life of an animal, is the best characterized [1, 2]. Other adult stem cells include neural stem cells (NSCs) [3], and the mesenchymal stem cells (MSCs) that give rise to osteoblasts, chondrocytes, adipocytes, and skeletal and smooth muscle myocytes [4]. In contrast to embryonic stem cells (ESCs), which can give rise to all cell types in an adult organism and are called pluripotent [5], HSCs, MSCs, and NSCs are termed multipotent. A number of recent studies have suggested that cells with more pluripotent features than HSCs, MSCs, or NSCs can be isolated from postnatal somatic tissues. Reyes [6] and Jiang [7] described a population of cells termed multipotent adult progenitor cells (MAPCs), which expand *in vitro* without obvious senescence, and can, at the clonal level, not only generate mesenchymal-lineage cells but also endothelium, hematopoietic cells, hepatocyte-like, and neuroectoderm-like cells *in vivo* and/or *in vitro*. Since the characterization of MAPCs, several other groups have described cells with similar abilities that can be isolated from bone marrow (human bone marrow stem cells (hBMSC), marrow-isolated adult multilineage inducible (MIAMI) cells, and pre-MSCs), umbilical cord blood (unrestricted somatic stem cells (USSCs)), placenta, muscle, and other tissues [8–13].

Despite the greater differentiation potential of the more pluripotent cells, such as MAPCs, compared with MSCs, it is not known whether MAPCs are different from classical postnatal MSCs. It is also not known how closely MAPCs resemble ESCs. We therefore applied principal components analysis (PCA), non-negative matrix factorization (NMF) and k-means clustering to analyze their transcriptomes and determine the relationship between MAPCs, MSCs and ESCs, using mouse and rat MAPCs and mouse MSCs and ESCs.

## Results

### Isolation and characterization of mouse and rat MAPCs

**Figure 1:**
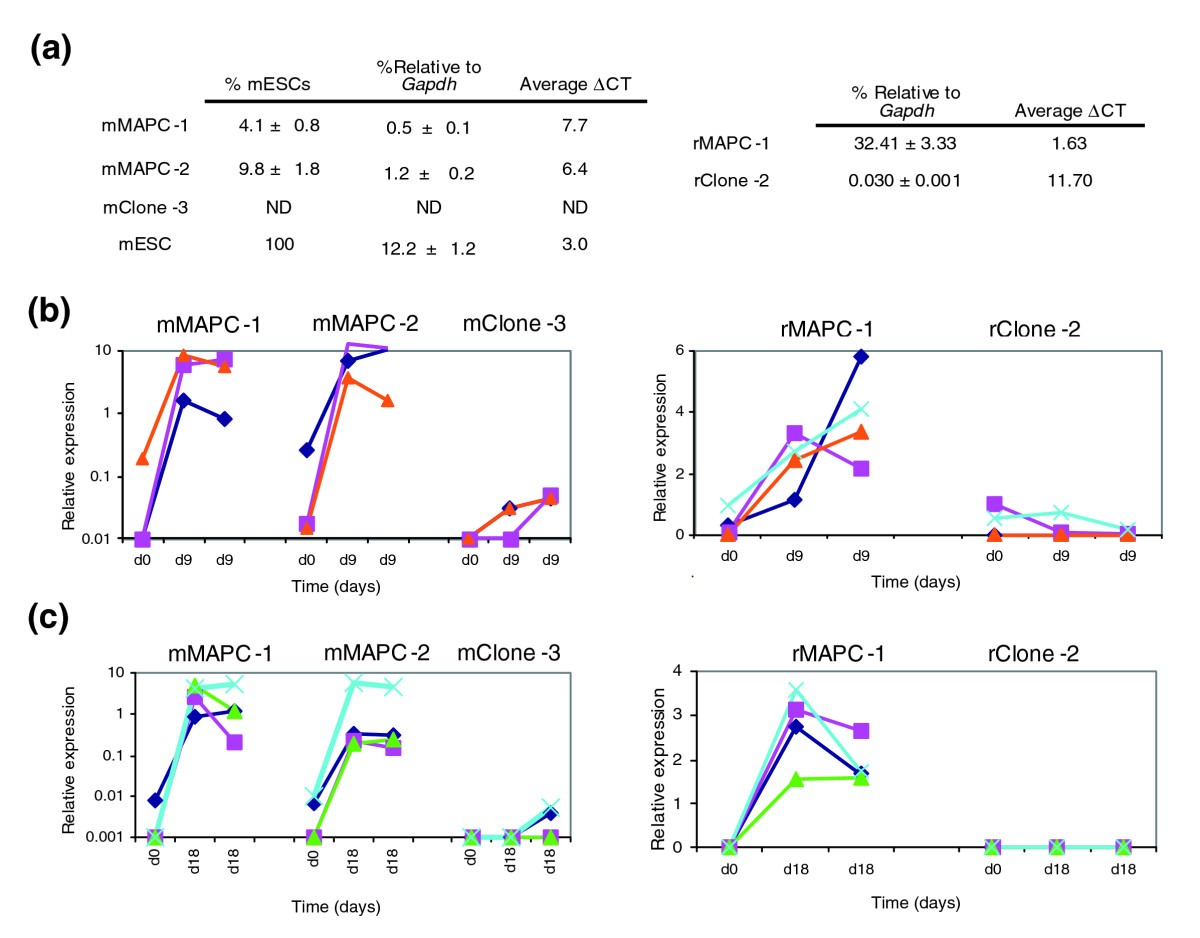
*Oct4* expression and endothelial-like and hepatocyte-like differentiation for mMAPC-1, mMAPC-2, mClone-3, rMAPC, and rClone-2. **(a)** The levels of *Oct4* (*Pou5f1*) mRNA in mouse (m, left) and rat (r, right) clones compared with those of *Gapdh* mRNA. The mouse clones are also compared with mESCs. ΔCT is difference in threshold cycles calculated as Oct4 CT - Gapdh CT. ND, not detected. **(b)** Endothelial-like differentiation. mRNA levels of endothelial markers in mouse (left panel) and rat (right panel) clones before and after differentiation, measured at day 9 in two independent differentiations of each clone. Levels are compared with those in universal mouse RNA and rat spleen RNA, respectively. Left panel: blue diamonds, Pecam (×10) (values shown were scaled by the factors in brackets); pink squares, Lyve1; orange triangles, vWF. Right panel: blue diamonds, VE-Cad (×100); pink squares, Flt-1; orange triangles, Flk-1; turquoise crosses, vWF (×100). **(c)** Hepatocyte-like differentiation. mRNA levels of hepatocyte markers in mouse and rat clones before and after differentiation. Levels are compared with levels in mouse hepatocytes and rat liver, respectively. Two representative differentiations measured at day 18 are shown. Left panel: blue diamonds, F2 (×100); pink squares, Tat, (×10^5^); green triangles, Afp (×10^-1^); turquoise crosses, Ttr (×10^3^). Right panel: blue diamonds, Afp (×100^-1^); pink squares, Alb, (×10^3^); orange triangles, Tat (×100); turquoise crosses, Ttr (×10^3^). See text for abbreviations.

**Figure 2:**
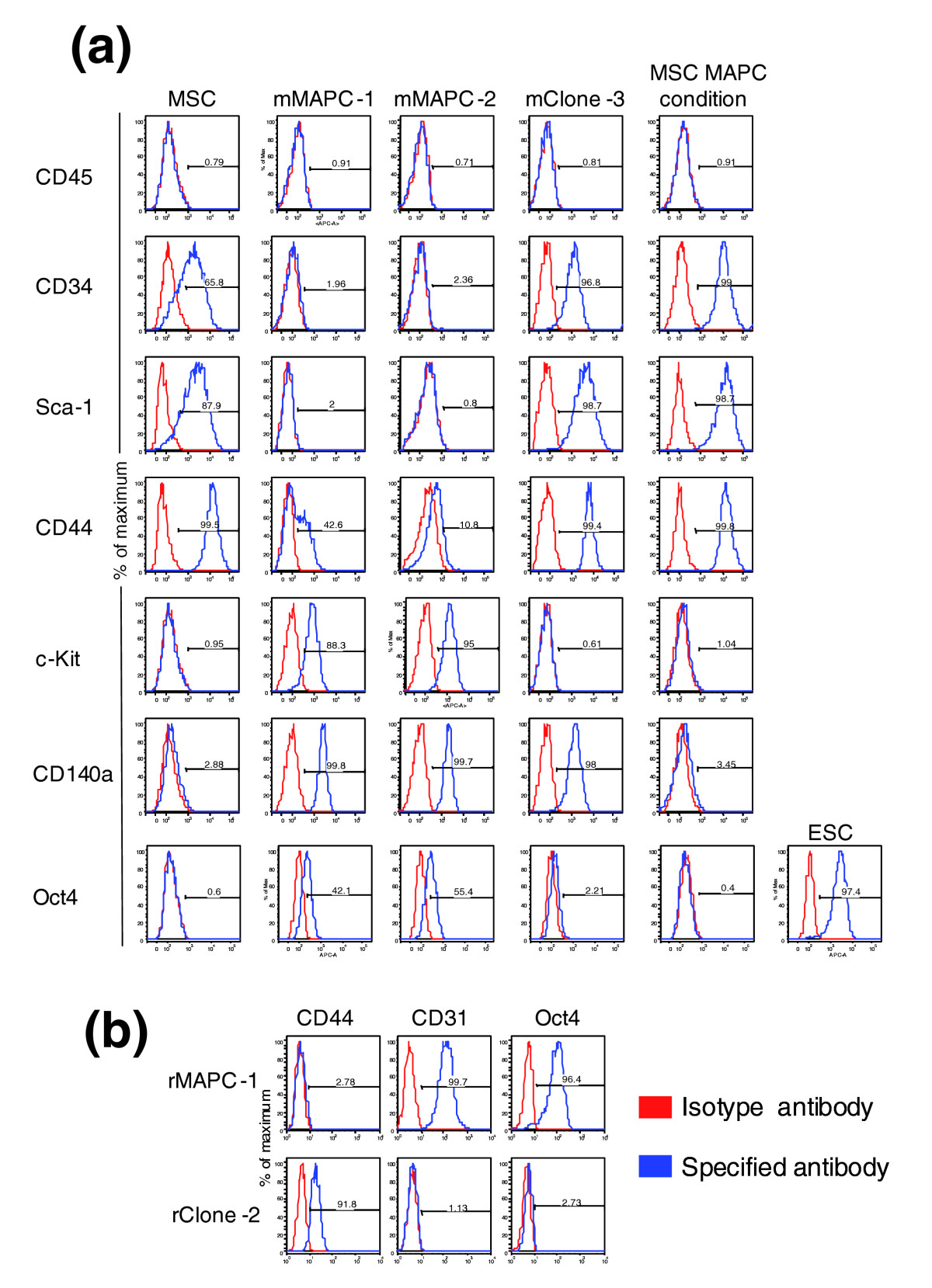
Cell-surface phenotype and Oct4 protein expression of mouse and rat clones evaluated by flow cytometry. **(a)** Flow cytometry results for mouse clones. From left to right: MSCs; mMAPC-1; mMAPC-2; mClone-3; and MSCs cultured in MAPC conditions. The histogram in each panel plots the number of cells (as a percentage of the maximum) on the vertical axis against the fluorescence intensity of the labeled antibody bound to the indicated protein (horizontal axis). The horizontal line on each histogram indicates the fluorescence range that contains the indicated percentage of cells positive for that protein. **(b)** Flow cytometry results for the rat clones rMAPC-1 and rClone-2.

Mouse (m) MAPCs and rat (r) MAPCs were isolated from marrow of C57BL/6-Tg-eGFP mouse and Fisher rats under 5% oxygen as recently described [14]. Two independently and clonally isolated mMAPC populations - mMAPC-1 and mMAPC-2 - expressed the mRNA for the transcription factor Oct4 at a level of 4-10% of the *Oct4* RNA levels in mouse ESCs or at a difference in threshold cycles (ΔCT) of 6 to 8 compared with the mRNA for glyceraldehyde-3-phosphate dehydrogenase (*Gapdh*) (Figure 1a). Similarly, rMAPCs isolated under these conditions expressed high levels of *Oct4* mRNA (ΔCT of 1 to 2 compared with *Gapdh* mRNA, Figure 1a). We have recently found that both mouse and rat MAPCs express Oct4 protein, which is localized in the nucleus [14]. However, whereas some clones isolated under MAPC culture conditions express Oct4 (mMAPC-1, -2 and rMAPC-1), other clones (mClone-3 and rClone-2 obtained in the same isolation as mMAPC-1 and rMAPC-1, respectively) do not express Oct4 (Figures 1a and 2b). The transcription factor Oct4 (Pou5f1) is expressed in early embryonic development and is essential for the maintenance of the pluripotent state of ESCs [15]. Although it has generally been accepted that Oct4 expression in adults is restricted to primordial germ cells, recent studies have shown that *Oct4* mRNA and/or protein can be detected in bone marrow cells following *in vitro* culture [7, 10, 16, 17], and may be expressed in some cells from bone marrow isolates [18, 19].

The cell-surface phenotypes of mouse mMAPC-1, mMAPC-2, and mMSC (obtained from D. Prockop, Tulane University) are shown in Figure 2a. MAPCs and MSCs are negative for the hematopoietic marker CD45. MAPCs, but not MSCs, express c-Kit and are negative for CD34 and Sca-1. Both populations express CD44, although MSCs express it at higher levels. Oct4 protein is homogeneously detected in both mMAPC populations at lower levels than in ESCs, but is absent in mMSCs (Figure 2a). Like the phenotype of mMAPCs, the phenotype of rMAPCs is homogeneous. rMAPCs express Oct4 and CD31, whereas rClone-2 expresses neither (Figure 2b). Karyotyping of the two mMAPC clones and rClone-2 showed that at least 65% of the cells were diploid, whereas 95% of the rMAPC-1 population was diploid. Both the mClone-3 and mMSCs contained less than 20% diploid karyotype.

The two mMAPC clones and the rMAPC clone were evaluated for their ability to differentiate *in vitro* towards endothelium-, hepatocyte- and neuroectoderm-like cells. Both mMAPC and rMAPC clones cultured for 9 days in the presence of vascular endothelial growth factor A (VEGF-A) showed a significant increase in transcript levels of lymphatic endothelial-associated genes (for example, *Lyve-1*) and endothelial markers (von Willebrand factor (vWF), CD31, vascular endothelial cadherin (VE-cadherin), and the VEGF receptors Flt-1 and Flk-1) (see Figure 1b). MAPC-derived progeny also acquired functional characteristics of endothelium as they form vascular tubes and take up acetylated low-density lipoprotein (ac-LDL) (A.L. and C.M.V., unpublished work). Moreover, we have evidence that the mMAPCs used here generate HSCs *in vivo* that reconstitute the lympho-hematopoietic system [20], and when grafted into the limbs of mice with limb ischemia induce significant recovery of perfusion and muscle function within 3 weeks, in part due to the incorporation of MAPC progeny into endothelium, smooth muscle and skeletal muscle in the ischemic limb (A.L. and C.M.V., unpublished work). Of note, we did not see formation of tumors from mMAPCs in these transplantation experiments. When MAPCs were cultured with bone morphogenetic factor 4 (BMP4), the fibroblast growth factors FGF2 and FGF8, hepatocyte growth factor (HGF), oncostatin M (OSM), and dexamethasone, a significant induction of the mRNAs for alfafetoprotein (*Afp*), transthyretin (*Ttr*), tyrosine aminotransferase (*Tat*), albumin (*Alb*) and coagulation factor 2 (*F2*) was seen (Figure 1c). MAPC-derived progeny also acquired functional characteristics of hepatocyte-like cells, as they secrete albumin and conjugate billirubin (K.A.P. and C.M.V., unpublished work). Finally, mouse or rat MAPCs cultured at low density in N2/B27 medium [21] express transcripts specific for neuroectoderm, including *Sox2*, *Sox1* and *Pax6*, as well as Sox2 and Pax6 protein (A.C., M.G. and C.M.V., unpublished work). These results together show that MAPCs have a much broader differentiation capacity compared with MSCs. Mouse MSCs differentiated into osteoblast and adipocyte progeny (as described by Peister *et al*. [22], data not shown).

### Transcriptome analysis of mMAPCs compared with MSCs and ESCs

**Figure 3:**
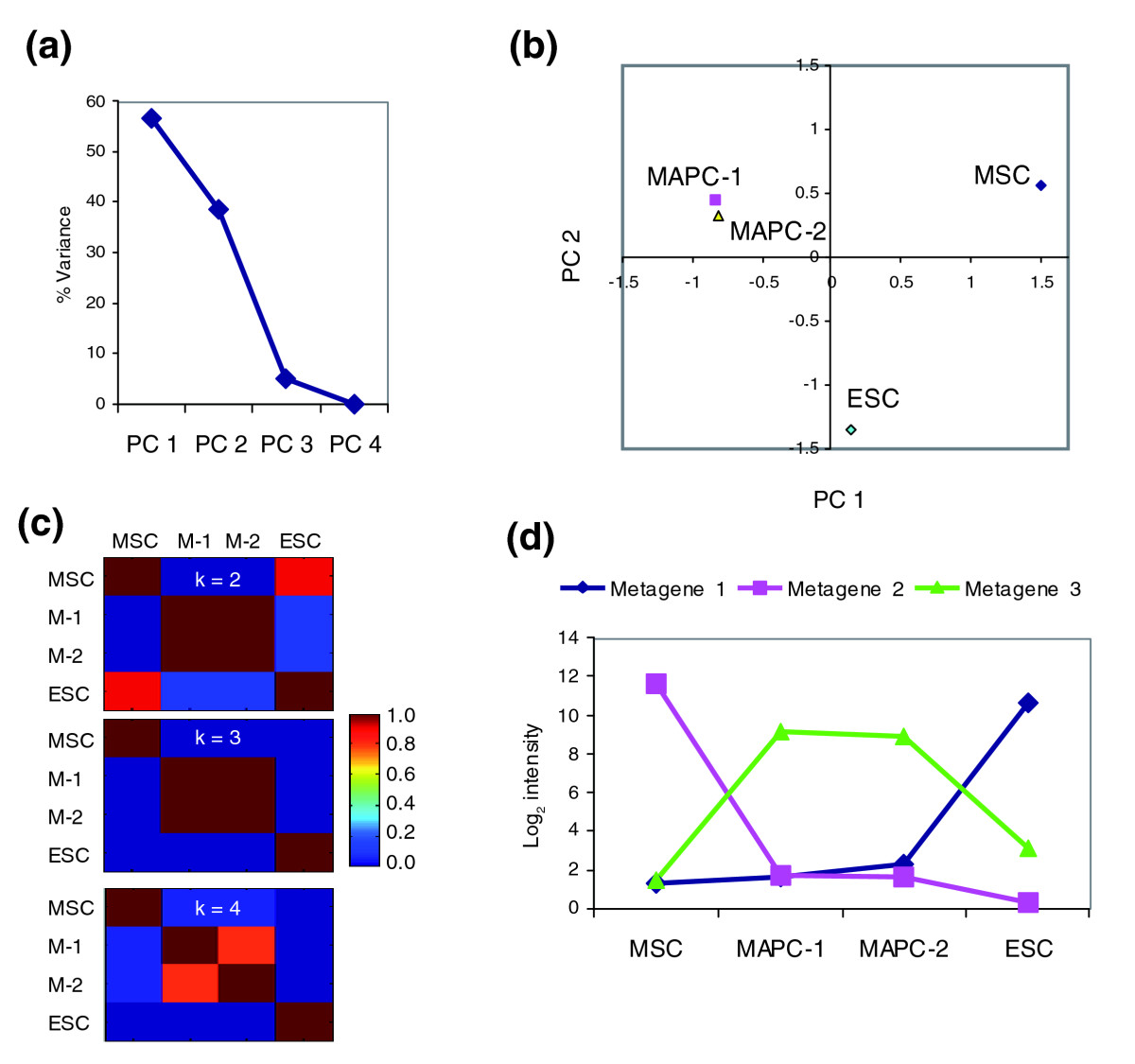
PCA and NMF analysis of the gene-expression data of mouse MSC, MAPC-1, MAPC-2 and ESC. **(a)** Percentage variation captured in each principal component (PC). PCs are ordered from 1 to 4 according to the percentage of the total variance they capture. **(b)** Samples plotted in the first two components' space. Distance of samples in the component space is indicative of similarity in expression profiles. **(c)** Consensus matrix from NMF. Model selection in NMF is based on a consensus matrix that contains the probability that a pair of samples is assigned to the same group. Probability values correspond to the colors in the key. M-1, MAPC-1; M-2, MAPC-2; **(d)** Metagene profiles from NMF plotted as logarithm of the probe set intensity.

In a first set of studies we compared the transcriptomes of the two mMAPC clones (mMAPC-1 and -2), C57BL/6 mouse MSCs, and C57BL/6 mouse ESCs. All cell populations were harvested during log-phase of expansion, that is, 2-3 days after subculturing, to avoid differences in expression data due to differences in cell-cycle state. Three samples of RNA for each cell type were collected at different passages for gene-expression profiling using Affymetrix Mouse 430 2.0 arrays. We observed little variation in gene expression over time, as the correlation coefficients of probe-set intensity values between the replicates were at least 0.98. The average of the expression levels from the three replicates were used for analysis and genes with twofold difference in expression levels between any pair of cell types (MAPC clones were treated individually) and with a false-discovery rate (FDR) of less than 0.5% (evaluated using significance analysis of microarrays (SAM) [23]) were considered as differentially expressed). This resulted in 9,702 differentially expressed transcripts. PCA was used to reduce the dimensionality of the expression dataset and represent it as a linear combination of two main orthogonal variables (principal components, PCs) [24, 25] (PCA is a dimensionality-reduction technique based on singular value decomposition of the covariance matrix that allows expression and visualization of genes or samples in a reduced components space defined by the PCs). Figure 3a shows that the first two PCs captured 95% of the total variance. Plotting samples in this two-PC-reduced dimensional space demonstrated clear separation of the mMAPC populations, ESCs, and MSCs into three distinct groups (Figure 3b).

We used NMF to group samples and genes according to the major patterns of expression in the dataset (metagenes) [26]. NMF is based on a linear decomposition of the expression data into two matrices with non-negative entries and allows representation of the expression data in a k-dimensional space where k is the number of groups or 'metagenes'. The optimal number of clusters is determined after a consensus matrix is built on the basis of the metagene expression pattern of each sample. We found that the best grouping, defined as the consensus matrix where probability entries are either 0 or 1, is with k = 3 groups or metagenes (Figure 3c). Through NMF, those samples were also separated into three groups: MSCs, mMAPCs, and ESCs. Correlation coefficients of genes to each metagene were calculated for gene clustering. The expression profiles of those three metagenes are shown in Figure 3d. Metagene 1 (consisting of 2,304 genes) is highly expressed in ESCs and low or not expressed in mMAPCs or MSCs; metagene 2 (consisting of 2,442 genes) is highly expressed in MSCs and low or not expressed in mMAPCs or ESCs; and metagene 3 (consisting of 1,551 genes) is highly expressed in mMAPCs but low in ESCs and MSCs.

**Figure 4:**
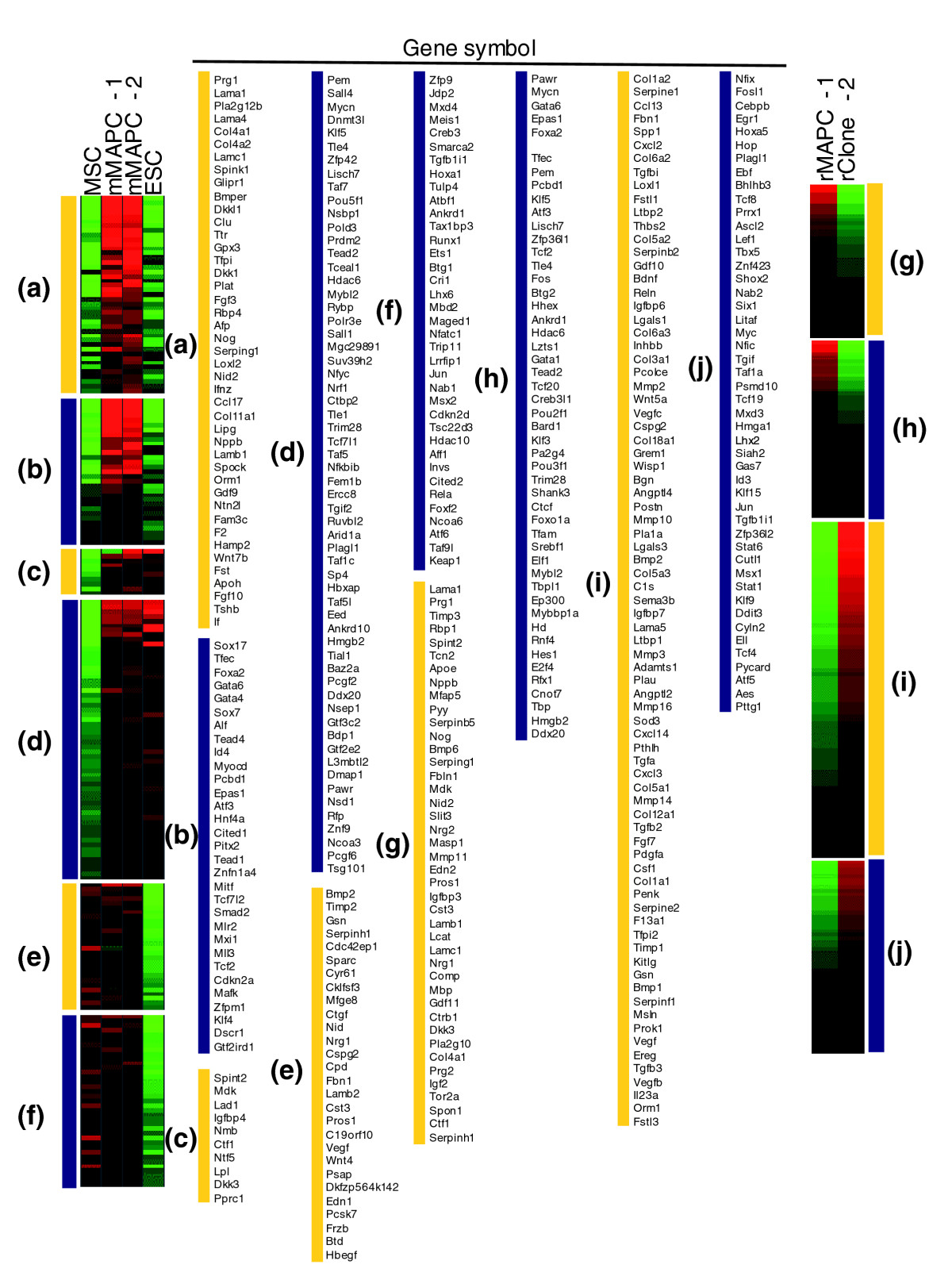
Genes for cell-surface and extracellular space proteins and transcriptional regulators clustered according to their expression profiles in the mouse MAPC/ESC/MSC comparison and in rat rMAPC-1 and rClone-2. **(a, b)** Genes expressed in mMAPCs at levels at least twofold higher than in ESCs and MSCs. **(c, d)** Genes expressed in mMAPC/ESCs at levels at least twofold higher than in MSCs. **(e, f)** Genes expressed in mMAPC/MSCs at levels at least twofold higher than in ESCs. **(g, h)** Genes expressed at levels at least twofold higher in rMAPC-1 than in rClone-2. **(i, j)** Genes expressed in rClone-2 at levels at least twofold higher than in rMAPC-1. The yellow bars indicate genes for proteins localized to the extracellular space; the blue bars indicate genes for transcriptional regulators. Red and green indicate higher and lower levels of expression, respectively.

**Figure 5:**
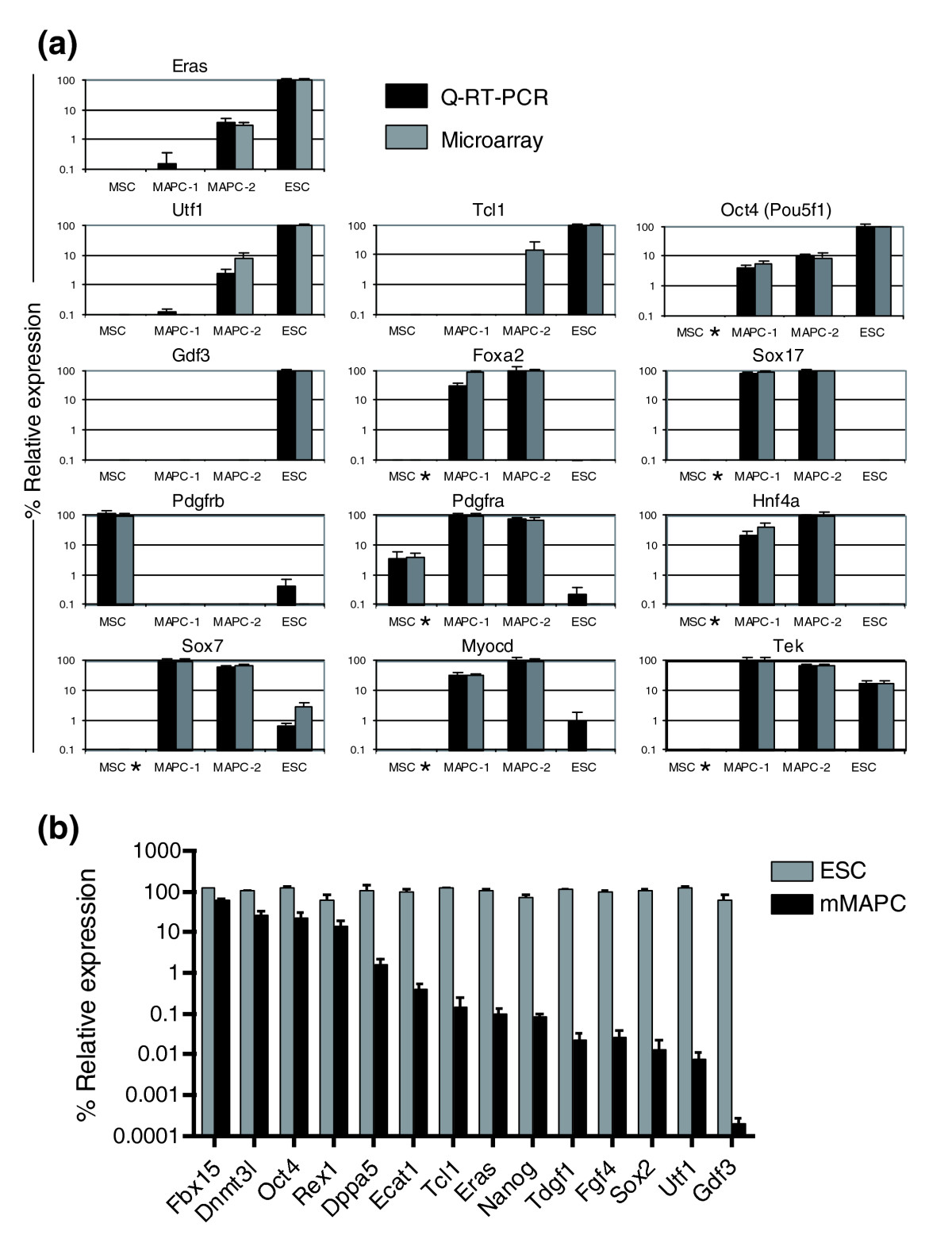
Q-RT-PCR validation of differentially expressed genes. **(a)** Comparison of relative expression levels of mRNA for the genes indicated by Q-RT-PCR on unamplified RNA (black bars) and Affymetrix microarray data (gray bars) (*n* = 3). Samples were normalized using *Gapdh* as the housekeeping gene and percent expression level was calculated with respect to the highest-expressing sample. The asterisk (*) indicates genes evaluated in mMSCs grown in the MAPC condition. **(b)** Relative mRNA expression levels of Ecats and other ESC-expressed transcripts in mMAPCs (black bars) and ESCs (gray bars) quantified using Q-RT-PCR. Three independent isolations of mMAPCs and ESC RNA for Q-RT-PCR were used to confirm microarray mRNA expression results. Samples were normalized to ESC expression levels and *Gapdh* mRNA levels were used for internal normalization.

Among the 1,551 genes in metagene 3, 546 genes were more than twofold differentially or uniquely expressed in mMAPCs (Figure 4a,b and Additional data file 1). mMAPCs expressed transcripts for a set of transcription factors that are expressed during specification to extraembryonic, primitive, or definitive endoderm during embryonic development. These include *Sox17*, *Foxa2*, *Gata6*, *Gata4*, *Sox7*, *Hnf4α*, *Cited1*, and *Tcf2*. Also expressed were transcripts of laminin *Lamb1*, the adaptor protein *Dab2* and other basement membrane components such as *LamA1*, *LamA4*, *Lamc1*, *Col4a1*, and *Nidogen 2*. Some of these genes (*Lamb1*, *Dab2*) are known to be induced by Gata6 [27, 28]. This expression pattern is also seen in primitive endoderm and extraembryonic endoderm cell lines (XEN cells) [29, 30], and can be induced in ESCs or in the inner cell mass (ICM) by knocking out or downregulating expression of *Nanog* [31–33]. This is consistent with the fact that even though mMAPCs express *Oct4* mRNA they do not express *Nanog*. In addition, mMAPCs also uniquely expressed transcripts of a small number of mesodermal transcription factors, such as *Tefc*, *Myocd*, *Pitx2*, and *Mitf*, at levels significantly higher than in MSCs and ESCs. A number of these genes were chosen for quantitative real-time PCR (Q-RT-PCR) on unamplified RNA for confirmation of the microarray results (Figure 5).

A set of 757 genes have expression levels in both mMAPCs and ESCs at least twofold higher than in MSCs (Figure 4c,d and Additional data file 1). We explored the levels of transcripts that are found to be specifically expressed in mouse ESCs in other studies; it should be noted that some of these genes might not be implicated in maintaining the ESC state. The degree of expression of such genes in MAPCs or MSCs *per se* also does not prove that one or the other cell populations is more related to ESC. An extensive search for ESC-specific transcripts was performed by Mitsui *et al*. [31] by *in silico* differential expression. This approach yielded a list of 20 genes that are enriched in ESCs, named ESC-associated transcripts (Ecats), corroborated *Oct4* and *Rex1* (*Zfp42*) as ESC markers, and identified *Nanog* as required for maintaining ESC pluripotency. Six out of 20 Ecats were expressed in both ESCs and mMAPCs (*Sall4*, *Dnmt3l*, *Dppa5*, *Fbxo15*, *Rex1* (*Zfp42*) and *Oct4* (*Pou5f1*)). Two Ecats (*Zfp296* and *Ecat6*) were expressed in mMAPCs and in MSCs, although at lower levels, whereas another two Ecats (*Eras*, *Utf1*) were expressed only at low levels in mMAPC-2 and not in mMAPC-1. This was seen in the microarray assay and was confirmed by Q-RT-PCR on unamplified RNA as shown in Figure 5a, whereas the final 10 Ecats (*Ecat1*, *Tcl1*, *Tdgf1*, *Nanog*, *Ecat8*, *Nr0b1*, *Gdf3*, *Map3k8 or* Est, *Hnrnpg-t* and *Brachyury* (T-box)) were not expressed in mMAPCs and their absence has been confirmed by Q-RT-PCR on multiple samples besides the ones used for microarray assay (Figure 5). Of the six Ecats expressed in both MAPC clones, *Dppa5* and *Fbxo15* are dispensable for maintaining ESC pluripotency [34, 35]. *Sall4*, one of the Ecats highly expressed in MAPCs, was recently described as essential for pluripotency in ESCs and early embryonic development through its direct regulation of *Oct4* transcription [36, 37]. Like the overexpression of *Oct4*, overexpression of *Sall4* in mouse ESCs directs them to a primitive endoderm fate when leukemia inhibitory factor (LIF) is withdrawn [36]. Of three additional genes recently implicated in self-renewal of mouse ESCs [38], *Tbx3* was expressed in mMAPCs at higher levels than in ESCs, *Dppa4* at low levels, and *Esrrb* expression was not detected in mMAPCs. The α6 integrin present in the 'stem cell signature' [39–41] was also expressed in both ESCs and mMAPCs. *Klf4* and *Mycn* (encoding a transcription factor related to c-Myc), which together with *Oct4* and *Sox2* can induce an ESC-like phenotype in murine fibroblasts, were expressed in both mMAPCs and ESCs [35]. Importantly, subsequent studies found that fibroblasts into which the four transgenes had been introduced could produce chimeric mice when *Nanog* expression was activated [42, 43]. A number of transcription factors involved in early embryonic development (*Pem*, *Klf5*, *Tead2* and *Sall1*), as well as *Lin28*, a gene downregulated during ESC differentiation [44], *Tex19*, a testis-specific transcription factor [45] and several members of the Bex family (some of them involved in neural differentiation - *Rex3*, *Bexl1*, and *Bex2* [46]) were coexpressed in mMAPCs and ESCs.

Genes highly expressed in ESCs at levels at least twofold those in MSCs and mMAPCs include genes for 93 transcription factors. One of these was *Sox2*, which, like *Nanog* and *Oct4*, is required for the pluripotent character of ESCs [47]. Genes such as *Fgf4* (a target gene for Oct4 and Sox2 [48]), *Nodal* and *Lefty* [49], which all encode well-known ESC-expressed secreted factors, were not expressed in mMAPCs or MSCs.

Eight hundred genes were coexpressed between MSCs and mMAPCs at levels at least twofold higher than in ESCs (Figure 4e,f and Additional data file 1). These included genes for 37 transcription factors, most of which play a role in early mesoderm development, including *Meis1*, *Hoxa1*, *Lhx6*, *Runx1*, and *Msx2*. A similar early endoderm phenotype is seen when mMAPCs are compared with ESCs using long-oligonucleotide arrays

To substantiate the results obtained using the Affymetrix platform and confirm that the genes associated with MAPCs and MAPCs/ESCs were not the result of the cell type to which they were compared (that is, MSCs) or of the array platform used, we compared the transcriptomes of mMAPC-1 and ESCs to an enriched, although not pure, neural progenitor population, namely neurospheres (NS) derived from embryonic day 11.5 mouse brain. This analysis was performed by hybridization on long-oligonucleotide arrays (National Institute on Aging (NIA) Mouse 44 K Microarray v2.1 slides; Whole Genome 60-mer oligo) in comparison with universal RNA. Genes with a twofold difference in expression and an FDR of less than 5% were considered to be differentially expressed. Using k-means clustering, we found that six clusters capture the major patterns in data variability among these samples.

**Figure 6:**
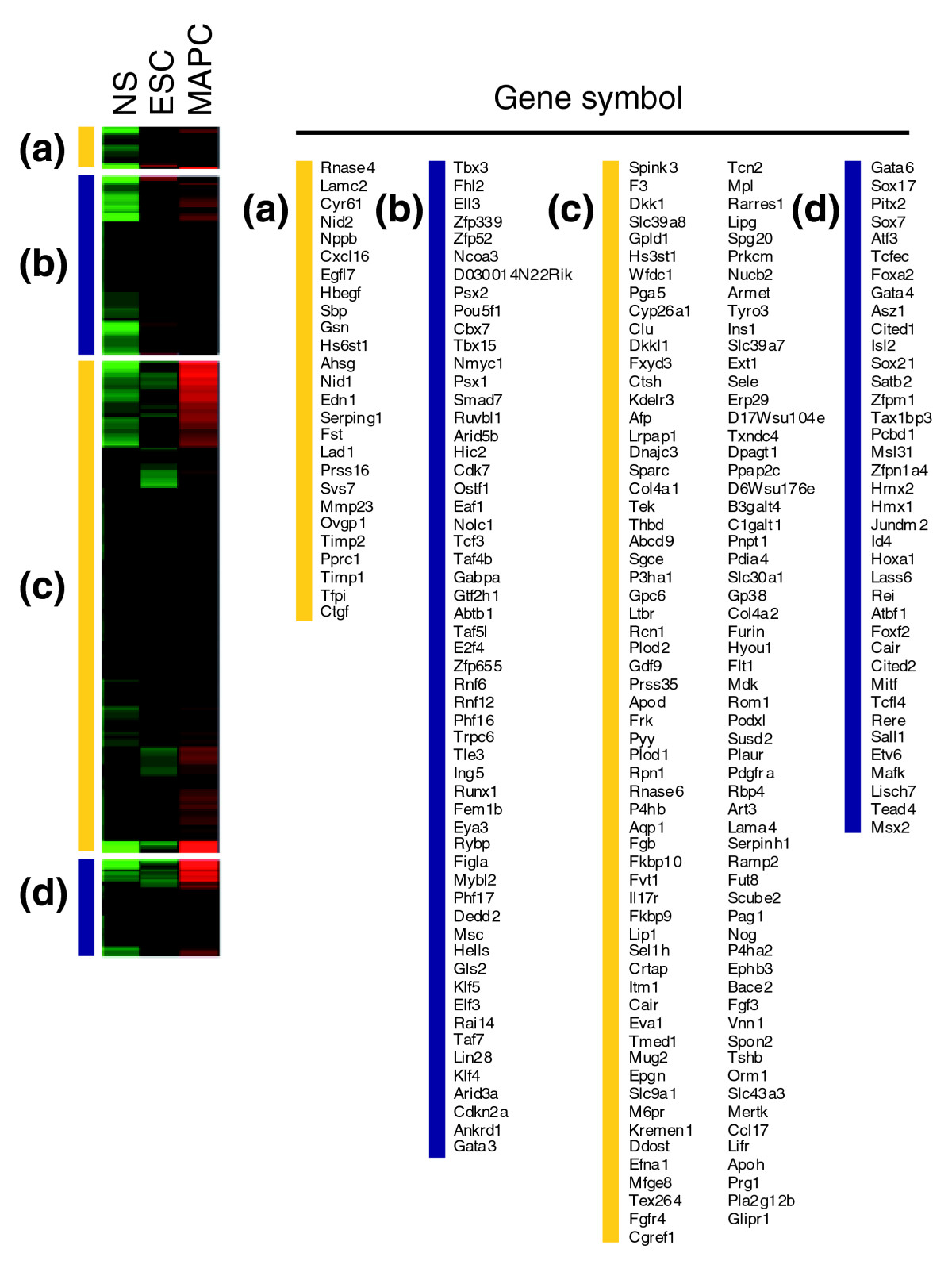
Genes for proteins localized to the extracellular space and for transcriptional regulators clustered according to expression profiles in MAPC/ESC/NS comparison. **(a, b)** Genes expressed at levels at least twofold higher in both MAPCs and ESCs than in NS (k-means clusters 2 and 4). **(c, d)** Genes expressed in MAPCs at levels at least twofold higher than in both ESCs and NS (k-means cluster 3). The yellow bars indicate genes whose products are localized to the extracellular space; the blue bars indicate genes for transcriptional regulators. Red and green indicate higher and lower levels of expression, respectively.

A cluster containing 775 genes expressed in mMAPCs at levels at least twofold higher than in both ESCs and NS was identified (Figure 6c,d and Additional data file 1), with many of these genes functioning or being transcribed during endoderm specification, including *Gata4*, *Gata6*, *Sox7*, *Sox17*, *Cited1*, *Lamb1*, *LamA1*, *LamA4*, *Col4a1*, *Dab1*, *Nidogen 2* and *Afp*. Of the 38 transcription factors enriched in mMAPCs, we found the mesodermal transcription factors *Pitx2* and *Mitf*, also identified in the ESC-MAPC-MSC comparison, to be most highly expressed in mMAPCs. Additional mesodermal transcription factors expressed in mMAPCs include *Hmx1*, *Hmx2*, *Hoxa1*, and *Msx2*. When the same analysis was done simply comparing genes differentially expressed between MAPCs and ESCs, without NS as a comparator, a similar expression pattern emerged. Within the genes expressed at more than twofold higher levels in MAPCs compared with ESCs and with an FDR of less than 5%, we again identified the genes functioning or being transcribed during endoderm specification as well as early mesodermal transcription factors (Additional data file 1).

Five hundred and thirty genes were expressed in mMAPCs and ESCs at levels at least twofold higher than in NS (Figure 6a,b and Additional data file 1). Of the 20 Ecats, three (*Dnmt3l*, *Fbxo15*, and *Sall4*) were more highly expressed in mMAPC-2 than in ESCs or NS, and four (*Oct4* (*Pou5f1*), *Rex-1* (*Zfp42*), *Dppa5*, and *Zfp296*) were highly expressed in mMAPC-2 and ESCs, although ESCs expressed these genes at levels at least twofold higher than mMAPCs. Low levels of *Utf1*, *Eras*, *Hnrnpg*, and *Gdf3* transcripts were found in mMAPC-2; the former two were confirmed by Q-RT-PCR (Figure 5a,b). Eight Ecats - *Ecat1*, *Ecat8*, *Nanog*, *Nr0b1*, *Brachyury*, *Map3k8* (EST), *Tcl1*, and *Tdgf1* - were not expressed at significant levels in mMAPCs, and *Ecat6* was not found on the NIA array. Expression of Ecat genes and other ESC-enriched genes in mMAPCs compared with ESCs is consistent with the Affymetrix microarray. When the analysis was done without NS as comparator, a similar picture emerged (Additional data file 1).

Among the 530 genes expressed in mMAPCs and ESCs at levels at least twofold higher than in NS, a number were also more highly expressed in ESCs and MAPCs than in MSCs in the Affymetrix analysis, including *Tbx3*, *Tbx15*, *Taf4b*, *Tcf3*, *Nmyc*, *Taf7*, *Klf4*, *Klf5*, *Klf8*, and the Oct4-regulated gene *Dppa2* [50]. Consistent with the ESC/MAPC/MSC analysis, *Dppa4*, *Lefty*, *Fgf4*, and *Nodal* were uniquely expressed in ESCs in the ESC/MAPC/NS analysis.

The agreement of the results obtained by both the Affymetrix and the NIA microarray platforms was corroborated by comparing clusters of genes expressed in MAPCs or coexpressed between MAPCs and another cell type (ESC, MSC, or NSC) obtained from both platforms. This analysis demonstrated that more than 65% of genes expressed in MAPCs in either analysis were identical, even though gene assignment to clusters was somewhat different in the ESC/MAPC/MSC versus ESC/MAPC/NS analysis as a result of the differences between MSCs and NS. One hundred and forty-nine genes found to be expressed at significantly higher levels in mMAPCs compared with NS and ESCs with the NIA array were also significantly expressed in MSCs falling into the MAPC/MSC cluster in the Affymetrix dataset. These include genes for mesodermal transcription factors such as *Hoxa1*, *Msx2*, and *Cited2*. Similarly, 132 genes coexpressed between MAPCs and ESCs at higher levels than in NS in the NIA dataset were not differentially expressed when compared to MSCs and were not assigned to the MAPC/ESC cluster in the MAPC/ESC/MSC comparison. Taking these factors into consideration, 349/550 and 257/379 genes more highly expressed in mMAPCs and in both mMAPCs and ESCs, respectively, in the NIA dataset were also more highly expressed in MAPCs, or showed similar expression in MAPCs and ESCs, in the Affymetrix dataset, and are therefore likely to represent the mMAPC molecular signature (Additional data file 1).

### The mesendodermal mMAPC signature is not induced by the culture medium

**Figure 7:**
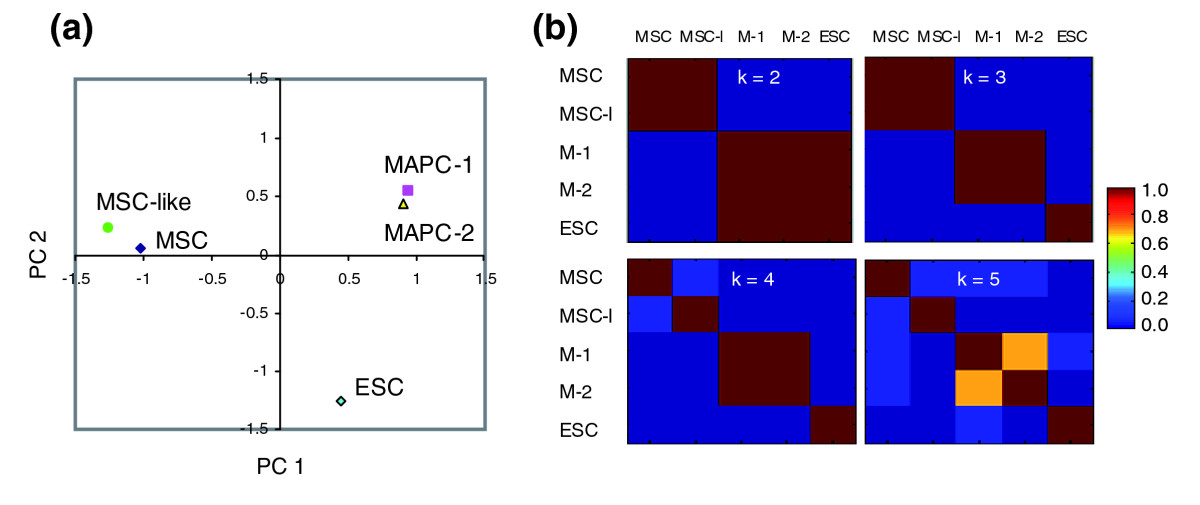
PCA and NMF analysis on the gene-expression data of MSC, MSC-like (MSC-*l*, mClone-3), MAPC-1 (M-1), MAPC-2 (M-2) and ESC. **(a)** PCA on all differentially expressed genes with samples plotted in the first two components' space. **(b)** Consensus matrix from NMF on all differentially expressed genes. See Figure 3 for an explanation of the key in (b).

One possible explanation for the differences in the mMAPC and MSC transcriptomes could be the differences in culture conditions used in their isolation and expansion (2% serum, platelet derived growth factor (PDGF)-BB, epidermal growth factor (EGF) and LIF for mMAPCs and 10% FCS plus 10% horse serum for MSCs). We therefore compared the transcriptomes of MAPCs, ESCs, and MSCs with that of mClone-3, which was isolated under conditions used to isolate mMAPC. mClone-3 did not express *Oct4* mRNA and differentiated poorly into endothelial or hepatocyte-like cells (Figure 1a-c). Moreover, we have shown that cells with low levels of Oct4 do not generate HSCs that can repopulate the lympho-hematopoietic system *in vivo* [20]. In contrast to mMAPC1 and mMAPC2, mClone-3 expresses the cell-surface antigens Sca-1 and CD44, but not c-Kit (Figure 2). Using PCA, the non-*Oct4*-expressing mClone-3 cells (named MSC-like cells) were shown to be very similar to MSCs but not to the *Oct4*-expressing mMAPCs (Figure 7a). The first two PCs accounted for 84% of the total variation, whereas the third component that discriminates between MSC-like cells and MSCs accounted for only 12% of the variation. NMF analysis similarly demonstrated that MSC-like cells are closely related to MSCs. The consensus matrixes obtained by grouping two or three groups gives elements of only 0 or 1, placing MSCs and MSC-like cells together in a group (Figure 7b). Seven hundred and forty-three genes were found to be more than fourfold differentially expressed between MSC and MSC-like cells, irrespective of their expression level in the other cell types (Additional data file 1). Forty-four of the genes more highly expressed in MSCs than in MSC-like cells were for transcription factors, of which most are regulators of mesoderm development (*Dlx5*, *Hoxc13*, *Sox11*, *Lhx6*, and *Dlx6*) but a few are regulators of neural development (*Emx2*) or endoderm/mesoderm development (*Gata6*). Likewise, rare regulators of endoderm (*Pcbd1*, whose protein is a dimerization cofactor of HNF1α) and ectoderm (*Sox2*, *Isl-1*) and more regulators of mesoderm development (*Gata2*, *Hoxa11*, *Mitf1*, *Sox5*, *Sox6* and *Cebp*δ), were more highly expressed in MSC-like cells than in MSCs. Neural cell adhesion molecule (*NCAM*) and cadherin 11 (*Cdh11*), which are expressed in MSCs, were not expressed in MSC-like cells, and *Nrcam*, *Pdgfra*, *CD24*, and *Cdh13* were expressed in MSC-like cells but not in MSCs. One thousand seven hundred and seventy-two (1,772) transcripts were expressed in both MSC and MSC-like cells at levels at least twofold higher than in mMAPCs and ESCs. Transcription factors in this list have known roles in development and morphogenesis of mesodermal tissue (Hox family members, *Runx2*, *Cebpβ*, *Pparγ*, and *Sox9*). Both MSC and MSC-like cells expressed transcripts for morphogens such as Bmp4, Bmp1, transforming growth factor beta 3 (TGFβ3), inhibin beta A (Inhba), PDGF-A, PDGF-C, and Wnt5a. Cell-surface markers coexpressed in MSCs and MSC-like cells included CD34, which is known to be expressed on murine MSCs, and Sca-1 and CD44, which, as mentioned above, discriminates between mMAPCs and MSC-like cells. Hence, although some differences could be detected in MSCs and MSC-like cells, they involve mainly regulators of mesoderm differentiation and specification, and did not involve genes that define either ESCs or MAPCs.

To further address the question of whether differences in culture conditions were responsible for differences in the ESC, MAPC, and MSC transcriptomes, we carried out PCA on subsets of genes reported to be upregulated by short-term culture under hypoxic conditions (16 or 24 hours at 1-1.5% O_2_) in mESCs or rat MSCs by Hu *et al*. [51] (44 genes) and Onhishi *et al*. [52] (135 genes), respectively. Neither of these two subsets of genes separates MAPCs, MSCs, and ESCs (Additional data file 2). It is notable that none of the endoderm-, mesoderm- or ESC-associated genes expressed in MAPCs is more highly expressed in MSCs kept under hypoxic conditions for 24 hours [52].

In a second set of studies, we evaluated the effect of culturing the MSCs obtained from Tulane University for 15 population doublings under MAPC conditions (5% O_2_ and MAPC medium). The cell-surface phenotype did not change significantly, and MSCs cultured under MAPC conditions remained negative for c-Kit and positive for CD44, Sca-1, and CD34 (Figure 2a). CD34 and Sca1 were more homogeneously expressed on mMSCs cultured under MAPC conditions, and more similar to mClone-3, than on mMSCs maintained in MSC culture conditions (Figure 2a). mMSCs cultured under MAPC conditions also did not express *Oct4* transcripts (as determined by Q-RT-PCR) or Oct4 protein (as determined by FACS analysis) (Figure 2b). Finally, using Q-RT-PCR, we determined whether genes highly expressed in MAPCs but not in MSCs (less than 0.1% of MAPC expression) were induced following culture for 14 days under MAPC conditions. Culture of mMSCs in MAPC conditions for 15 doublings did not induce expression of *Sox17*, *Sox7*, *HNF4α*, *Tek*, or *Myocd* (Figure 5a). Similarly, no change was detected in the expression of *Pdgfra* (CD140a) at the mRNA or protein level (Figures 2a and 5a). *Pdgfra* is, however, one of the genes more highly expressed in mClone-3 cells than in MSCs, at levels near those seen in mMAPC-1 and mMAPC-2. This suggests that isolation and long-term maintenance of cells under MAPC rather than MSC conditions might be responsible for some of the differences in gene expression between MAPCs and MSCs. We therefore identified the set of genes whose expression pattern was similar to that of *Pdgfra*. Only 146 genes were highly correlated or anti-correlated with *Pdgfra* (0.85 absolute correlation) suggesting that these genes might be upregulated or downregulated, respectively, as a result of MAPC culture conditions (Additional data file 1). Interestingly, this list of genes includes *Klf4* and a few endodermal markers such as *Lam4*, *Col4*, and *Tcf2*. Removal of this subset of genes from the analysis did not significantly alter sample clustering by either PCA or NMF (Additional data file 2).

### Signature of rat rMAPCs is similar to that of mouse mMAPCs

Finally, we evaluated the transcriptomes of one clone of rat MAPCs (rMAPC-1) expressing high levels of *Oct4* mRNA and protein and one clone of rat cells that expressed 1,000-fold lower levels of *Oct4* mRNA and differentiated poorly into endothelium or hepatocyte-like cells (rClone-2), even though it was isolated and cultured under MAPC conditions (Figure 1a-c). The transcriptome was analyzed using Affymetrix Rat 230A arrays, which contain 4,699 known rat sequences and 10,467 expressed sequence tags. Of the 2,869 probes differentially expressed with an FDR of 1.75%, 1,285 were annotated as unique genes (Additional data file 1). Data from different species were not compared directly, because only relative gene expression (ratio of two samples), instead of the absolute abundance of mRNA, is obtained. Thus, we compared genes differentially expressed between rMAPC-1 and rClone-2 to those differentially expressed between mMAPC-1, mMAPC-2, and MSCs. We identified a panel of 585 genes that were differentially expressed in both the mouse and rat analysis using Affymetrix arrays. Of these genes, 86% show the same trend in expression in both datasets; that is, they were either more highly expressed in the high-Oct4-expressing rMAPC-1, mMAPC-1, and mMAPC-2, or in cells that do not express *Oct4* - rClone-2 and mouse MSCs (Additional data file 1). The correlation coefficient of 0.7 when we plot the relative gene-expression levels in the two datasets further confirms the similarities in the MAPC molecular signature in both rodent species (Additional data file 2).

A significant number of genes differentially expressed between rMAPC-1 and rClone-2 encoded transcription factors. As in mMAPCs, we saw an enrichment in *Oct4*-expressing rMAPCs (rMAPC-1) of transcription factors and extracellular matrix components found in trophectoderm, primitive endoderm, and definitive endoderm (*Foxa2*, *Gata6*, *Gata4*, *HNF1β*, *Cited1*, *Tcf2*, *Lamb1*, *Lama1*, *Lamc1*, *Col4a1*, and *Nidogen 2*), as shown in Figure 4g,h and Additional data file 1. High-*Oct4*-expressing rMAPCs also expressed transcription factors involved in mesodermal development, similarly to the expression seen in mMAPCs, as well as transcription factors known to be involved in early development, which were also found in the set of genes expressed by both mMAPCs and mESCs. Similarly to our findings in mClone-3 (MSC-like cells) isolated using the same conditions as for MAPC, the low-*Oct4*-expressing rClone-2 cells chiefly expressed genes involved in mesoderm development, particularly those for mesenchymal regulators and markers, including *Cebpβ*, *Hoxa5*, *Shox2*, *CD105*, tensile collagens, and *CD44* (Figure 4j). CD44 expression was also observed by flow cytometry (Figure 2 and Additional data file 1). rClone-2 also expressed a large number of soluble factors, including those in the TGFβ family, different VEGFs, Wnt, PDGF, and the hematopoietic modulators stem-cell factor (SCF) and colony-stimulating factor (CSF), which are characteristic of stromal cells such as MSCs. Some of these were also found in mClone-3 and mMSCs (Figure 4i and Additional data file 1). The similarity in expressed gene profile between rClone-2 and mouse MSCs shows that cells with similar characteristics to MSCs can be isolated from both mouse and rat using MAPC culture conditions, and further supports the notion that the major differences in the expressed gene profile between MAPCs and MSCs is not caused by differences in the media used to isolate and/or expand MAPCs.

## Discussion

The comparative transcriptome analysis described here demonstrates a unique molecular signature for MAPCs compared with MSCs. Although both MAPCs and MSCs are isolated from bone marrow as an adherent cell population, we previously showed that the differentiation potential of MAPCs far exceeds that of MSCs [6, 7]. Consistent with the notion that MAPCs are distinct from MSCs, we have used MAPCs from two rodent species to demonstrate that the MAPC transcriptome differs significantly from that of MSCs or MSC-like cells obtained under the same culture conditions used for MAPC isolation. The MSC-like cells do not express Oct4 and differentiate poorly towards endothelium- and hepatocyte-like cells. Furthermore, mouse cells that express very low levels of Oct4 also cannot generate HSCs when grafted *in vivo* [20].

We also show that a subset of genes expressed in MAPCs is also expressed in pluripotent ESCs. For instance, compared with MSCs, MAPCs express *Oct4* transcripts, and Oct4 protein is present in more than 90% of MAPCs [14]. In addition, MAPCs express nine other Ecat genes, which were identified by Mitsui *et al*. [31] to be ESC-associated, even though not all of these genes have been found to play a role in maintaining ESC properties. Moreover, MAPCs express Klf4 and a Myc family transcription factor, Mycn - which are expressed in diverse tissues and cell types but have been shown to be required with Oct4, Sox2 and, indirectly, Nanog for conferring ESC-like characteristics on cultured fibroblasts [35, 42, 43]. MAPCs also express the ESC self-renewal regulators Tbx3 and Dppa4 [38]. Obviously, expression of this subset of genes does not prove that MAPCs are more closely related to ESCs than to MSCs at the global transcriptional level. However, this analysis provides a list of candidate regulators of MAPC self-renewal that will need to be validated using functional studies to elucidate their role in self-renewal and differentiation potential of adult stem cells.

MAPCs do not, however, express a key transcription factor for ESC pluripotency - Nanog. During development, loss of *Nanog* expression from cells that express *Oct4* is associated with the expression of *Gata6* and restriction of cells in the ICM to primitive endoderm [33]. *Sall4*, a gene recently found to directly regulate *Oct4* expression in ESCs and which is involved in epiblast and primitive endoderm specification *in vivo*, is highly expressed in MAPCs [36, 37]. Consistent with these known molecular interactions, we show here that the transcriptome of mouse and rat MAPCs resembles that of extraembryonic endoderm cell lines, parietal endoderm, and visceral endoderm [29]. However, MAPCs also express a number of mesodermal, and to a lesser extent ectodermal, transcripts. Remarkably, a similar conclusion can be drawn when MAPCs are compared with ESCs and NS, or to ESCs only, using a distinct microarray platform and clustering algorithm. It should be noted that despite the fact that mMAPCs do not express *Nanog*, MAPCs express 84 genes that are presumed transcriptional targets in ESCs for Nanog alone or both Nanog and Oct4 [53]. Whether the absence of *Nanog* expression in mMAPCs might be compensated for by transcription factor activity of Stat3, Mycn, Klf4, Klf5, and Klf8, which bind to some of the Nanog target genes, will need to be determined. The classification of MAPCs, MSCs, and ESCs by PCA and NMF using their respective transcriptomes thus correlates with their differentiation capacity.

More important, this study demonstrates for the first time that clonal isolation under identical conditions can yield cells with transcriptional and functional characteristics of MAPCs or cells with characteristics of MSCs. Although the analysis cannot answer the question of whether MAPCs exist in bone marrow or are a culture-induced phenomenon, we show that genes found to be more highly expressed in ESCs or MSCs following short-term exposure to hypoxia [51, 52] are not similarly upregulated in cells isolated and cultured under MAPC conditions; hence acute hypoxia-responsive genes are not responsible for differences in the transcriptomes of MAPCs and either MSCs or ESCs. Furthermore, no change in cell-surface phenotype, or expression of *Oct4* or other genes expressed at significantly higher levels in MAPCs compared with MSCs, was seen when cells isolated and characterized as MSCs were cultured for 14 days in MAPC culture conditions. It should be noted that the gene for one of the PDGF receptors, namely *Pdgfra*, which is not expressed in MSCs, is expressed in mClone-3 at levels near those found in mMAPCs, but was not induced in MSCs maintained for 14 days under MAPC conditions. This might suggest that isolation and/or long-term maintenance of cells under the specific MAPC culture conditions, rather than MSC conditions, may influence the transcriptome. However, we demonstrated that only 146 genes were highly correlated or anti-correlated with *Pdgfra*. Removal of this set of genes from the dataset did not influence the clustering of MAPCs, MSCs, and ESCs by either NMF or PCA, further demonstrating that differences between MSCs and MAPCs are not simply the result of differences in culture conditions.

One possible explanation for the isolation of MAPC-like or MSC-like cells, is that apart from MSCs, the more pluripotent Oct4-positive stem cells remain in the bone marrow during development, as suggested by Kucia *et al*. [18] and Anjos-Afonso and Bonnet [13]. However, the CD45^-^Lin^-^ Sca1^+^ cells identified in bone marrow by Kucia *et al*. [18] coexpress *Nanog* mRNA and protein, and express significantly lower levels of endodermal as well as mesodermal and ectodermal transcripts. The SSEA1^+^ CD45^-^ Lin^-^ CD31^-^ population sorted directly from bone marrow by Anjos-Afonso and Bonnet [13] also express *Oct4* and *Nanog* mRNA and protein. Like MAPCs, these cells differentiated at the clonal level into cells of the three germ layers *in vitro* and to multiple mesodermal cell types *in vivo*. It should be noted that only when MAPC culture conditions were used could the cell phenotype and differentiation capabilities be maintained *in vitro*. The primitive endoderm-like phenotype of MAPCs may be explained by heterogeneous expression of *Oct4* and *Nanog* in cells from bone marrow, or by their prolonged expansion in the presence of PDGF and EGF in the culture medium. MAPCs express the tyrosine kinase receptor Pdgfra, which is expressed in primitive endoderm in mouse embryos before gastrulation [54]. Numerous transcriptional targets of PDGF signaling were discovered by Chen *et al*. [55]. Some of these target genes (*Tead4*, *Kit*, *CD9*, *Gfpt1*, and *Mrpl38*) were more highly expressed in MAPCs than ESCs, MSCs, or NS.

It is also possible that the *in vitro* culture and clonal expansion under the conditions described here induces reprogramming or dedifferentiation of stem cells in bone marrow. However, the finding that MSC-like cells that do not express Oct4 are also isolated under these conditions from mouse and rat indicates that not all bone marrow cells can be reprogrammed.

## Conclusion

Regardless of their origin, the isolation of cells with pluripotent capacity from postnatal somatic tissues yields another source of stem cells to study and to compare self-renewal and differentiation mechanisms in stem cells, in cell-based therapies, or *in vitro* drug screening. Here we demonstrate by using multivariate analysis of gene-expression data that MAPCs are different from MSCs and ESCs at the global transcriptome level and that their expressed gene profile strongly correlates with their *in vitro* differentiation capacity, as they express transcripts of early endoderm and mesoderm. MAPCs also express a subset of ESC-associated transcripts, some of which are known to play a role in the maintenance and self-renewal of ESCs. MAPCs, however, do not express Nanog or Sox2, two key transcription factors in the ESC pluripotency network. Future knockdown and overexpression studies will be needed to demonstrate that expression of genes known to impart pluripotency in ESCs are responsible for the broader differentiation capacity of MAPC compared with other adult stem cells. The molecular characterization of MAPCs compared with multipotent stem cells as well as ESCs should aid significantly in the future prospective isolation and culture of more pluripotent adult stem cells. This study should also allow the cross-comparison of different adult stem-cell populations with presumed broader differentiation ability, in order to elucidate the genes that confer these properties on them.

## Materials and methods

### Cell culture and RNA collection for gene arrays

C57BL/6 mice carrying the gene for enhanced green fluorescent protein (eGFP) were provided by I.L. Weissman (C57BL/6-Tg-eGFP) and by M. Okabe, Osaka University, Japan (C57BL/6TgN (act-EGFP) ObsC14-Yo1-FM1310 mice). MAPCs were isolated as described by Breyer *et al*. [14]. Briefly, bone marrow was plated in 60% low glucose DMEM (Gibco BRL, Grand Island, NY), 40% MCDB-201 (Sigma, St Louis, MO) containing 10 ng/ml mEGF (Sigma), hPDGF-BB (R&D Systems, Minneapolis, MN) and 1000 units/ml mLIF (Chemicon International, Temecula, CA), 2% screened FCS (Hyclone, Logan, UA, Lot ANL19977), 1 × selenium-insulin-transferrin-ethanolamine (SITE), 0.2 mg/ml linoleic acid-bovine serum albumin (LA-BSA), 0.8 mg/ml BSA (all from Sigma), 1 × chemically defined lipid concentrate (Gibco) and 55 μM 2-mercaptoethanol (Gibco), 100 units of penicillin, 1,000 units of streptomycin (Cellgro, Herndon, VA) in a humidified, 5% O_2_ and 6% CO_2_, 37°C incubator. After 4 weeks, CD45^+^ and Ter119^+^ cells were depleted using a MACS separation CS column (Miltenyi Biotec, Auburn, CA), and cells replated at 10 cells/well. Expansion was done by trypsinizing (0.05% trypsin, Cellgro) the cells and replating them every 2 days at a density of 100-200 cells/cm^2^. Cells at population doubling of more than 80 were used for three RNA collections for gene arrays and differentiation assays which were done over a span of 10 passages (25 to 30 population doublings). MAPC-1 and MSC-like cells were generated from C57Bl/6TgN (act-EGFP) ObsC14-Yo1-FM1310 mice and MAPC-2 from C57Bl/6-Tg-eGFP mice.

Mouse MSCs (C57BL/6) passage 5 were obtained from the Tulane University Center for Gene Therapy where they were isolated and shown to differentiate into bone and fat cells as described by Peister *et al*. [22]. They were thawed and cultured using medium and serum as recommended by the center and as described by Peister *et al*. [22]. Briefly, cells were cultured at a plating density of 100 cells/cm^2^ in IMDM (Gibco) with 2 mM L-glutamine (Gibco), 100 U/ml penicillin, 100 μg/ml streptomycin, 0.25 μg/ml amphotericin B (Gibco), 10% screened FBS (Atlanta Biologicals, Lawrenville, GA, Lot C0105) and 10% equine serum (Hyclone, Lot AQH24495) in a humidified, 21% O_2_, 5% CO_2_, 37°C incubator. As recommended, a first passage was done 1 day after thawing and subsequently cells were passaged every 6 days. RNA samples for gene arrays were taken 3 days after passaging with plates seeded at 300 cells/cm^2^ during three consecutive passages (10 population doublings).

Mouse ESCs (C57BL/6) passage 12 were maintained on irradiated mouse embryonic fibroblasts (MEFs) in high glucose DMEM with sodium pyruvate (Gibco), 15% FBS (Hyclone; Lot ANC18367), 2 mM L-glutamine, 100 units of penicillin, 1,000 units of streptomycin, 100 μM 2-mercaptoethanol and 1,000 units/ml mLIF in a humidified, 21% O_2_, 5% CO_2_, 37°C incubator. Cells were passaged every 2 days and RNA for gene arrays was collected during three passages (12 population doublings) from ESCs plated without MEFs.

Neuroepithelial cells were isolated from the forebrains of E11.5 C57BL/6 embryos by dissecting telencephalons and dissociating them into a near single-cell suspension in DMEM media. Approximately 1-2 × 10^5^ cells were plated into 6-well plates containing DMEM/F-12 (Gibco) and N-2 plus medium (R&D Systems) supplemented with 20 ng/ml mEGF and mFGF-2 (R&D Systems). Neurospheres (NS) were passaged every 4 days by centrifuging for 5 min at 40 *g* and partially dissociating the NS by pipetting up and down. Total RNA was prepared from passage 2 NS (eight population doublings).

Rat MAPCs were isolated as described [14]. Briefly, Fisher rat BM cells were plated on 60% low-glucose DMEM, 40% MCDB-201, 1 × insulin-transferrin-selenium (ITS, Sigma), 1 × LA-BSA, 0.05 × 10^-6^ M dexamethasone (Sigma), 10^-4^ M ascorbic acid 3-phosphate, 100 units penicillin, 1,000 units streptomycin, 55 μM 2-mercaptoethanol, 2% FBS (HyClone; Lot ANL19977), 10 ng/ml hPDGF-BB, 10 ng/ml mEGF, and 1,000 units/ml mLIF in a humidified, 5% O_2_ and 6% CO_2_, 37°C incubator. After 4 weeks, CD45^+^ cells were depleted using a MACS separation CS column, and cells replated at 10 cells/well. Expansion was done by trypsinizing (0.05% trypsin) the cells and replating them every 2 days at a density of 200 cells/cm^2^. Cells at population doubling grater than100 were used for three RNA collections for gene arrays (20 population doublings).

### Cell surface phenotype and intracellular Oct4 staining

#### Cell-surface phenotype

Cells were collected by trypsinization, washed with PBS containing 3% FBS, and blocked for 10 min with 5% rat serum and anti-CD16/CD32 antibody. After washing, cells were incubated for 30 min at 4°C in 3% FBS containing conjugated antibodies. Cells were washed once and resuspended in PBS 3% FBS plus propidium iodide and analyzed by flow cytometry.

Antibodies for mouse cell-surface markers were: CD45-APC, Sca1-PE, CD44-PE, c-Kit-APC, CD140a-APC (eBioscience, San Diego, CA), CD34-APC (eBioscience). Antibodies for rat cell-surface markers were CD44-PE and CD31-FITC (all antibodies except indicated otherwise were from BDPharmingen, San Diego, CA).

#### Intracellular Oct4 staining

Cells were collected by trypsinization, washed twice with PBS and fixed in 4% paraformaldehyde for 10 min at room temperature. After washing twice with PBS, cells were permeabilized with 0.1% (w/v) saponin (Sigma), 0.05% (w/v) sodium azide in PBS (SAP buffer) and blocked for 5 min in SAP buffer plus 10% donkey serum. After blocking, cells were incubated for 15 min at room temperature in SAP buffer plus anti-Oct3/4 antibody clone N19 (Santa Cruz Biotechnology, Santa Cruz, CA) and washed twice with SAP buffer. Cells were incubated for 15 min at room temperature with secondary antibody Cy5-labeled anti-goat (Jackson Immunoresearch, West Grove, PA) diluted in SAP buffer, washed twice with SAP buffer and resuspended in PBS for analysis by flow cytometry.

### Karyotyping

Two days after cells were seeded, they were exposed to 0.1 μg/ml colcemid (Sigma) for 3 h, harvested, washed, and exposed to 75 mM KCl hypotonic solution for 5 min. Hypotonic solution was washed out by centrifugation and cells were fixed in 10 ml methanol/acetic acid (3:1) fixative for 5 min. Fixed cells were collected by centrifugation and resuspended in 0.2 ml methanol/acetic acid (3:1) fixative. Cells were dropped onto a hot glass slide. Chromosomes were stained using Wright's stain. Twenty chromosomal spreads were counted per population.

### MAPC differentiations

#### Endothelial differentiation

Mouse and rat MAPCs, as well as mClone-3 and rClone-2, were plated on day 0 in their respective growth medium at 60,000 cells/cm^2^ on fibronectin-coated 12-well plates. On day 1, medium was switched completely to differentiation medium in low-glucose DMEM/MCDB-201 (60:40) containing 10 ng/ml hVEGF-A (R&D Systems), 1 × ITS, 1 × LA-BSA, 10^-8^ M dexamethasone, 10^-4^ M ascorbic acid 3-phosphate, 100 units penicillin, 1,000 units streptomycin and 55 μM 2-mercaptoethanol. For mouse MAPCs, differentiations were done in the absence of FCS. For rat MAPCs, FCS was maintained at 2%. RNA samples were collected on day 9 for analysis.

#### Liver differentiation

Mouse and rat MAPCs, as well as mClone-3 and rClone-2, were plated at 50,000 cells/cm^2^ on matrigel-coated 12-well plates using low-glucose DMEM/MCDB-201 (60:40) containing 2% FBS, 0.25 × ITS, 0.5 × LA-BSA, 0.1 × 10^-6^ M dexamethasone, 10^-4^ M ascorbic acid 3-phosphate, 100 units penicillin, 1,000 units streptomycin, and 55 μM 2-mercaptoethanol. The growth factor used varied over time: 50 ng/ml hBMP-4 and 10 ng/ml hFGF2 from day 1 to day 6, 10 ng/ml mFGF-8b and 10 ng/ml hHGF from day 6 to day 9; and 10 ng/ml human oncostatin M and 10 ng/ml hHGF from day 9 to day 18 (all cytokines and growth factors from R&D systems). A 75% media change was done every 3 days and RNA samples were collected on day 18.

#### Neuroectoderm differentiation

Rat or mouse MAPCs were plated at 1,500/cm^2^ and 2000/cm^2^, respectively, on fibronectin-coated glass coverslips placed in a 6-well plate; the medium used was Neurobasal-A medium and DMEM/F12 (1:1) supplemented with B27 (0.5×), N2plus (0.5×), BME (0.1 mM) and L-glutamine (0.2 mM). A 60% medium change was performed every other day and RNA samples were collected on day 7.

### RNA isolation and Q-RT-PCR

Total RNA from undifferentiated and differentiated cells was extracted using the RNeasy microkit (Qiagen, Valencia, CA). mRNA was reverse transcribed using Superscript III reverse transcriptase (Invitrogen, Carlsbad, CA) and cDNA underwent 40 rounds of amplification (ABI PRISM 7700, Perkin Elmer/Applied Biosystems, Foster City, CA) as follows: 40 cycles of a two-step PCR (95°C for 15 sec, 60°C for 60 sec) after initial denaturation (95°C for 10 min) with 1 μl DNA solution, 1 × TaqMan SYBR Green Universal Mix PCR reaction buffer (Applied Biosystems). Primers used for amplification are shown in Table 1. mRNA levels were normalized using the housekeeping gene *GAPDH* and compared with mRNA levels in mouse universal RNA (Ambion, Austin, TX), Fisher rat spleen for endothelial-like differentiation, mouse hepatocytes (Ambion) or Fisher rat total liver for hepatocyte-like differentiation, and mouse embryonic brain E15 RNA or rat embryonic brain E17 RNA (both from Gentaur, Brussels, Belgium) for neuroectoderm differentiations.

**Table 1:** Primers used for determination of mRNA expression levels by Q-RT-PCR

Gene	Forward primer	Reverse primer
**Mouse**		
*Gapdh*	TGCACCACCAACTGCTTAG	GATGCAGGGATGATGTTC
*Oct4 (Pou5f1)*	CCAATCAGCTTGGGCTAGAG	CCTGGGAAAGGTGTCCTGTA
*Pecam*	GTCATGGCCATGGTCGAGTA	CTCCTCGGCGATCTTGCTGAA
*Lyve-1*	AGGAGCCCTCTCCTTACTGC	ACCTGGAAGCCTGTCTCTGA
*vWF*	GCCAAAGATCTGGAACAGTGT	GATGGAGAGGTTACACATCTC
*F2*	CAGCTATGAGGAGGCCTTTG	TCACACCCAGATCCATAGCA
*Tat*	TTAAGTCCAATGCGGACCTC	GCTCTGTGAATTCCACGTCA
*Afp*	GCCCTACAGACCATGAAACAAG	GTGAAACAGACTTCCTGGTCCT
*Ttr*	CTTTGCCTCTGGGAAGACC	CAGAGTCGTTGGCTGTGAAA
*Foxa2*	CCCGGGACTTAACTGTAACG	TCATGTTGCTCACGGAAGAG
*Hnf4a*	GGTCAAGCTACGAGGACAGC	ATGTACTTGGCCCACTCGAC
*Sox7*	CTTCAGGGGACAAGAGTTCG	CCATGACTTTCCCAGCATCT
*Sox17*	CACAACGCAGAGCTAAGCAA	TTGTAGTTGGGGTGGTCCTG
*Myocd*	CTGTGTGGAGTCCTCAGGTCAAACC	GATGTGTTGCGGGCTCTTCAG
*Pdgra*	ACGTTCAAGACCAGCGAGTT	CCTCCAGCATGGTGATACCT
*Pdgfrb*	CACCTTCTCCAGTGTGCTGA	GGAGTCCATAGGGAGGAAGC
*Tek*	AAGCATGCCCATCTGGTTAC	GCCTGCCTTCTTTCTCACAC
*Fbx15*	GGCCTTGAATGGAGAACTGA	TCAAACCACCCTAGGTCTGC
*Dnmt3l*	CCTGGTGAAGAACTGCCTTC	GCAAAGTGAGCTGCACAGAG
*Rex1*	CCTGCACACAGAAGAAAGCA	TCAGTCTGTCGAGGGCTCTT
*Dppa5*	CAGTCGCTGGTGCTGAAATA	TCCATTTAGCCCGAATCTTG
*Eras*	CCCTGCTTGTCCATGAGATT	TGGTAACTTGGTCGGAGAGG
*Utf1*	GGCCATACCTTCGAATCCTC	GGTTTGGTCGAAGGAACCTC
*Tdgf1*	GCATCCTACGAGGGAGTTGA	CACTGTGCTTGGCTGAAGAA
*Tcl1*	TTCCTCTCTGGGTGTTCAGG	ATCCCACACATTCCCTTTCA
*Ecat1*	GAATGCCTGGAAGATCCAAA	AAATCTCAGCTCGCCTTTCA
*Nanog*	GAGTGTGGGTCTTCCTGGTC	GAGGCAGGTCTTCAGAGGAA
*Gdf3*	CGCAGGACTTATGCTACGTG	CTGGGCCATGGTCAACTT
*Fgf4*	CCGACGAGTGTAAATTCAAAGAA	GGAAGTGGGTTACCTTCATGG
*Sox2*	ACCAGCTCGCAGACCTACAT	AGTGGGAGGAAGAGGTAACCA
*Sox1*	CACAACTCGGAGATCAGCAA	TGTAATCCGGGTGTTCCTTC
*Pax6*	TCAGACCTCCTCATACTCGTGCA	TGTAGGTATCATAACTCCGCCCA
**Rat**		
*Gapdh*	TGCACCACCAACTGCTTAG	GATGCAGGGATGATGTTC
*Oct4 (Pou5f1)*	CTGTAACCGGCGCCAGAA	TGCATGGGAGAGCCCAGA
*Vecad*	GGCCAACGAATTGGATTCTA	GTTTACTGGCACCACGTCCT
*Flt-1*	TGGCCAGAGGCATGGAGT	TCGCAAATCTTCACCACATTG
*Flk-1*	CCAAGCTCAGCACACAAAAA	CCAACCACTCTGGGAACTGT
*vWF*	CCCACCGGATGGCTAGGTATT	GAGGCGGATCTGTTTGAGGTT
*Afp*	ACCTGACAGGGAAGATGGTG	GCAGTGGTTGATACCGGAGT
*Alb*	CTGGGAGTGTGCAGATATCAGAGT	GAGAAGGTCACCAAGTGCTGTAGT
*Tat*	AACCTCAGCACCAATGTTCC	TCTTCAGAGCACCCTGGACT
*Ttr*	CAGCAGTGGTGCTGTAGGAGTA	GGGTAGAACTGGACACCAAATC
*Sox2*	AACCCCAAGATGCACAACTC	CCGGGAAGCGTGTACTTATC
*Otx1*	GTTCGCAAAGACTCGCTACC	CCGGAGACGACTTCTTCTTG
*Blbp*	CCAGCTGGGAGAAGAGTTTG	TTTCTTTGCCATCCCACTTC
*Pax6*	GTCCATCTTTGCTTGGGAAA	TAGCCAGGTTGCGAAGAACT

### Affymetrix microarray sample and data processing

Samples of 100 ng of total cellular RNA isolated from mouse MSCs, mClone-3, mMAPC-1 and -2, and ESCs were double amplified and labeled with the Two-Cycle Target Labeling and Control Reagents kit P/N 900494 (Affymetrix, Santa Clara, CA) following the manufacturer's protocol. Samples of 200 ng of total cellular RNA from rat rMAPC-1 and rClone-2 were double amplified and labeled to generate labeled cRNA for hybridization. The first round of *in vitro* transcription-based, linear amplification was performed using the RiboAmp OA RNA Amplification Kit (Arcturus, Mountain View, CA) followed by labeling with the Enzo Bioarray HighYield RNA Transcript Labeling Kit (Enzo Diagnostics, Farmingdale, NY) according to the manufacturers' instructions. Samples were hybridized to Affymetrix mouse 430 2.0 chips or rat Expression Set 230A chips, washed, and scanned at the University of Minnesota Affymetrix Microarray Core Facility as described in the Affymetrix GeneChip Expression Analysis Technical Manual. CEL files were loaded into GeneData Expressionist Refiner (GeneData, San Francisco, CA) to assess overall quality and obtain condensed single intensity values per probe set using the Microarray Analysis Suite Statistical algorithm (MAS 5.0). The mean of mean intensity values for all chips was used to normalize the mean intensity of each chip. A probe set intensity value threshold of 20 and 25 for mouse and rat chips, respectively, was determined in GeneData Expressionist Analyst according to the absent/present calls from MAS 5.0. The average of the expression levels from the three replicates were used for further analysis. Probes with at least twofold differential expression in one group with respect to any other group were considered as differentially expressed. The statistical significance of differential expression was determined using SAM [23]. Probes were annotated using the Web-based Affymetrix NetAffx analysis tool [56] and Ingenuity Pathway Analysis (Ingenuity, Mountain View, CA).

### NIA microarray sample and data processing

Total RNA was extracted from mMAPC-1, ESCs, and neurospheres using Trizol reagent (Invitrogen). ESCs were passaged for a short time on gelatin-coated dishes to remove feeder cells. Samples of 2.5 μg total RNA from MAPCs, ESCs, and NS were labeled with Cy3-CTP dye and a universal mouse reference RNA (Stratagene, La Jolla, CA) was labeled with Cy5-CTP dye using a fluorescent linear amplification kit (Agilent Technologies, Palo Alto, CA) [57]. Two biological replicates were used for hybridizations. cRNA from MAPCs, ESCs, and NS was added to a universal reference cRNA and hybridized to a NIA Mouse 44 K Microarray v2.1 slide (Whole Genome 60-mer oligo; Agilent 012799). Microarrays were hybridized and washed following Agilent protocol G4140-90030 (Agilent 60-mer oligo microarray processing protocol; SSC Wash, V1.0) and scanned using an Agilent DNA microarray scanner. Images from scanned microarrays were analyzed for feature intensity using Agilent Feature Extractor A7.5.1 software [57]. Cy3-CTP-labeled MAPC, ESC, and NSC cRNA samples were normalized to Cy5-CTP labeled universal reference cRNA. NIA Array Analysis Tool software [58] was used for analysis of variance (ANOVA), averaging of replicates, and annotation of probe IDs. A twofold difference in expression between one sample and any other sample was considered significant.

### Sample- and gene-clustering algorithms

The log-transformed and mean-centered intensity values of significant differentially expressed genes were utilized in PCA using software from Spotfire (Spotfire, Cambridge, MA) based on the algorithm described by Alter *et al*. [24]. NMF and consensus matrix were applied to the log-transformed intensity values of significant differentially expressed genes in the Affymetrix dataset using the algorithms implemented by Brunet *et al*. for Matlab [26]. Gene clustering was performed by calculating the correlation coefficients of each gene to metagenes, and genes were assigned as correlated to a metagene profile if the correlation coefficient was greater than 0.75 or anti-correlated if it was smaller than -0.75. Profile correlation coefficients to *Pdgfra* were calculated on genes with consistent twofold differential expression among MAPC (group 1) and non-MAPC (group 2) condition culture samples (for example, each sample from group 1 vs each sample from group 2). Owing to the nature of the two-channel data obtained from the NIA microarray, we decided to perform k-means clustering on this dataset [59] instead of NMF. This was done on log-transformed and mean-centered intensity values of differentially expressed genes using Spotfire.

The raw and processed microarray data in this paper have been deposited in the Gene Expression Omnibus (GEO) database [60] and have been assigned the accession numbers GSE5947 and GSE6933.

## Additional data files

The following additional data are available with this paper online. Additional data file 1 contains 14 tables that list various aspects of the stem-cell transcriptomes: supplementary Table 1 lists genes expressed in MAPC at levels at least twofold higher than in MSC and ESC; supplementary Table 2 lists genes expressed in MAPC and ESC at levels at least twofold higher than in MSC; supplementary Table 3 lists genes expressed in MAPC and MSC at levels at least twofold higher than in ESC; supplementary Table 4 lists genes expressed in MAPC at levels at least twofold higher than in ESC and NS; supplementary Table 5 lists genes expressed in MAPC and ESC at levels at least twofold higher than in NS; supplementary Table 6 lists genes expressed in MAPC at levels at least twofold higher than in ESC in NIA microarrays; supplementary Table 7 lists genes expressed in ESCs at levels at least twofold higher than in MAPC in NIA microarrays; supplementary Table 8 contains a MAPC gene cluster comparison between Affymetrix and NIA microarrays; supplementary Table 9 lists genes differentially expressed between MSCs and MSC-like cells; supplementary Table 10 lists genes correlated with Pdgfra with twofold differential expression between all MAPC conditions cultured cells vs ESCs and MSCs; supplementary Table 11 lists genes anti-correlated to Pdgfra with a twofold differential expression between all MAPC conditions cultured cells vs ESCs and MSCs; supplementary Table 12 lists genes with at least twofold higher expression in rMAPC-1 than in rClone-2; supplementary Table 13 lists genes with at least twofold higher expression in rClone-2 than in rMAPC-1; supplementary Table 14 lists common differentially expressed genes in both mMAPC vs MSC and rMAPC-1 vs rClone-2 comparisons. Additional data file 2 contains three figures showing analyses of hypoxic and Pdgfra correlated genes and correlation between mouse and rat MAPC expressed gene profile.: supplementary Figure 1 shows PCA of MSC, MSC-like (MSC-*l*, mClone-3), MAPC-1 (M-1), MAPC-2 (M-2) and ESC on reported ESC and MSC hypoxia upregulated genes; supplementary Figure 2 shows PCA and NMF analysis of MSC, MSC-like (MSC-*l*, mClone-3), MAPC-1 (M-1), MAPC-2 (M-2) and ESC on differentially expressed genes minus genes correlated or anticorrelated to Pdgfra; supplementary Figure 3 shows the correlation of fold difference on common differentially expressed genes between mMAPCs vs MSCs and rMAPC-1 vs rClone-2.

### Electronic supplementary material

Additional data file 1: Supplementary Table 1 lists genes expressed in MAPC at levels at least twofold higher than in MSC and ESC; supplementary Table 2 lists genes expressed in MAPC and ESC at levels at least twofold higher than in MSC; supplementary Table 3 lists genes expressed in MAPC and MSC at levels at least twofold higher than in ESC; supplementary Table 4 lists genes expressed in MAPC at levels at least twofold higher than in ESC and NS; supplementary Table 5 lists genes expressed in MAPC and ESC at levels at least twofold higher than in NS; supplementary Table 6 lists genes expressed in MAPC at levels at least twofold higher than in ESC in NIA microarrays; supplementary Table 7 lists genes expressed in ESCs at levels at least twofold higher than in MAPC in NIA microarrays; supplementary Table 8 contains a MAPC gene cluster comparison between Affymetrix and NIA microarrays; supplementary Table 9 lists genes differentially expressed between MSCs and MSC-like cells; supplementary Table 10 lists genes correlated with Pdgfra with twofold differential expression between all MAPC conditions cultured cells vs ESCs and MSCs; supplementary Table 11 lists genes anti-correlated to Pdgfra with a twofold differential expression between all MAPC conditions cultured cells vs ESCs and MSCs; supplementary Table 12 lists genes with at least twofold higher expression in rMAPC-1 than in rClone-2; supplementary Table 13 lists genes with at least twofold higher expression in rClone-2 than in rMAPC-1; supplementary Table 14 lists common differentially expressed genes in both mMAPC vs MSC and rMAPC-1 vs rClone-2 comparisons. (XLS 3 MB)

Additional data file 2: Supplementary Figure 1 shows PCA of MSC, MSC-like (MSC-*l*, mClone-3), MAPC-1 (M-1), MAPC-2 (M-2) and ESC on reported ESC and MSC hypoxia upregulated genes; supplementary Figure 2 shows PCA and NMF analysis of MSC, MSC-like (MSC-*l*, mClone-3), MAPC-1 (M-1), MAPC-2 (M-2) and ESC on differentially expressed genes minus genes correlated or anticorrelated to Pdgfra; supplementary Figure 3 shows the correlation of fold difference on common differentially expressed genes between mMAPCs vs MSCs and rMAPC-1 vs rClone-2. (PDF 88 KB)

## Authors’ original submitted files for images

Below are the links to the authors’ original submitted files for images. Authors’ original file for figure 1 Authors’ original file for figure 2 Authors’ original file for figure 3 Authors’ original file for figure 4 Authors’ original file for figure 5 Authors’ original file for figure 6 Authors’ original file for figure 7 Authors’ original file for figure 8
