# Supplementary material for: Comparative transcriptome analysis of embryonic and adult stem cells with extended and limited differentiation capacity
Source: Genome Biol. 2007 Aug 6;8(8):R163. doi: 10.1186/gb-2007-8-8-r163 (PMC2374994; doi:10.1186/gb-2007-8-8-r163)

Supplementary Figures

Supplementary Figure 1. PCA of MSC, MSC-like (MSC-*l*, mClone-3), MAPC-1 (M-1), MAPC-2 (M-2) and ESC on reported ESC and MSC hypoxia upregulated genes.

A. PCA on 44 genes reported to be upregulated in ESC under short-term acute hypoxia [52] with samples plotted in the first two components space
B. PCA on 135 genes reported to be upregulated in MSC under short-term acute hypoxia [53] with samples plotted in the first two components space

Supplementary Figure 2. PCA and NMF analysis on ESC and MSC hypoxia upregulated genes of MSC, MSC-like (MSC-*l*, mClone-3), MAPC-1 (M-1), MAPC-2 (M-2) and ESC on genes correlated or anticorrelated to Pdgfra.

A. PCA on all differentially expressed genes minus 146 Pdgfra correlated or anticorrelated genes with samples plotted in the first two components space
B. Consensus matrix from NMF on all differentially expressed genes minus 146 Pdgfra correlated or anticorrelated genes

Supplementary Figure 3. Correlation of fold difference on common differentially expressed genes between mMAPCs vs MSCs and rMAPC-1 vs rClone-2.

Supplementary Figure 1

A

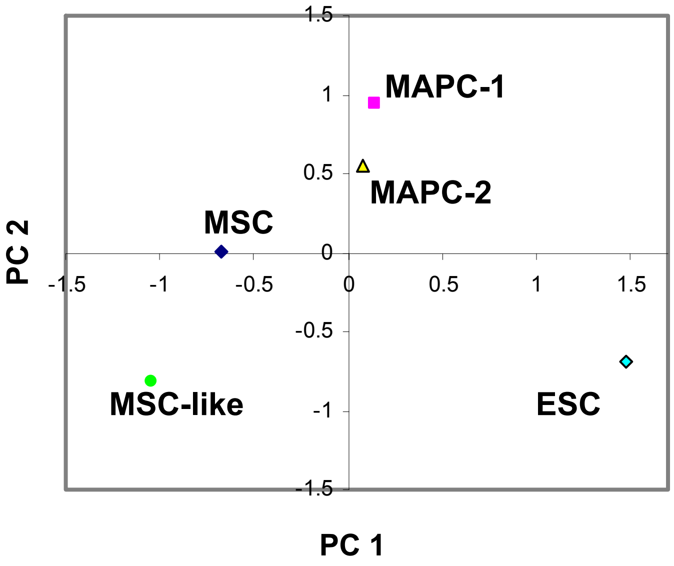

B

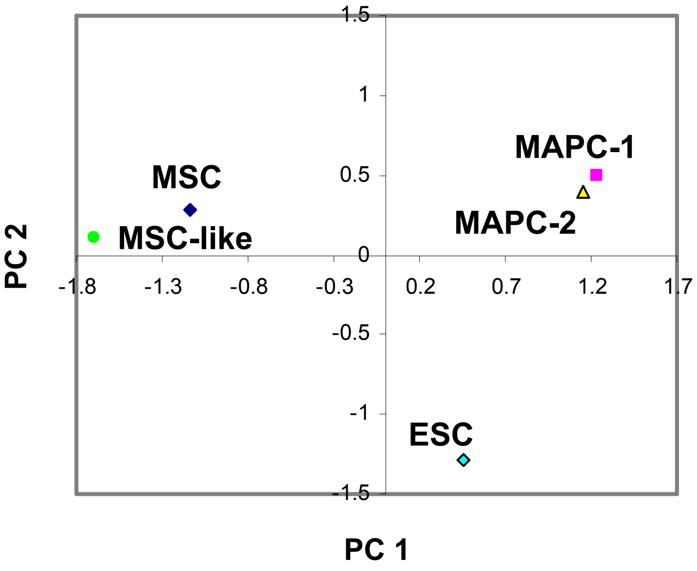

Supplementary Figure 2

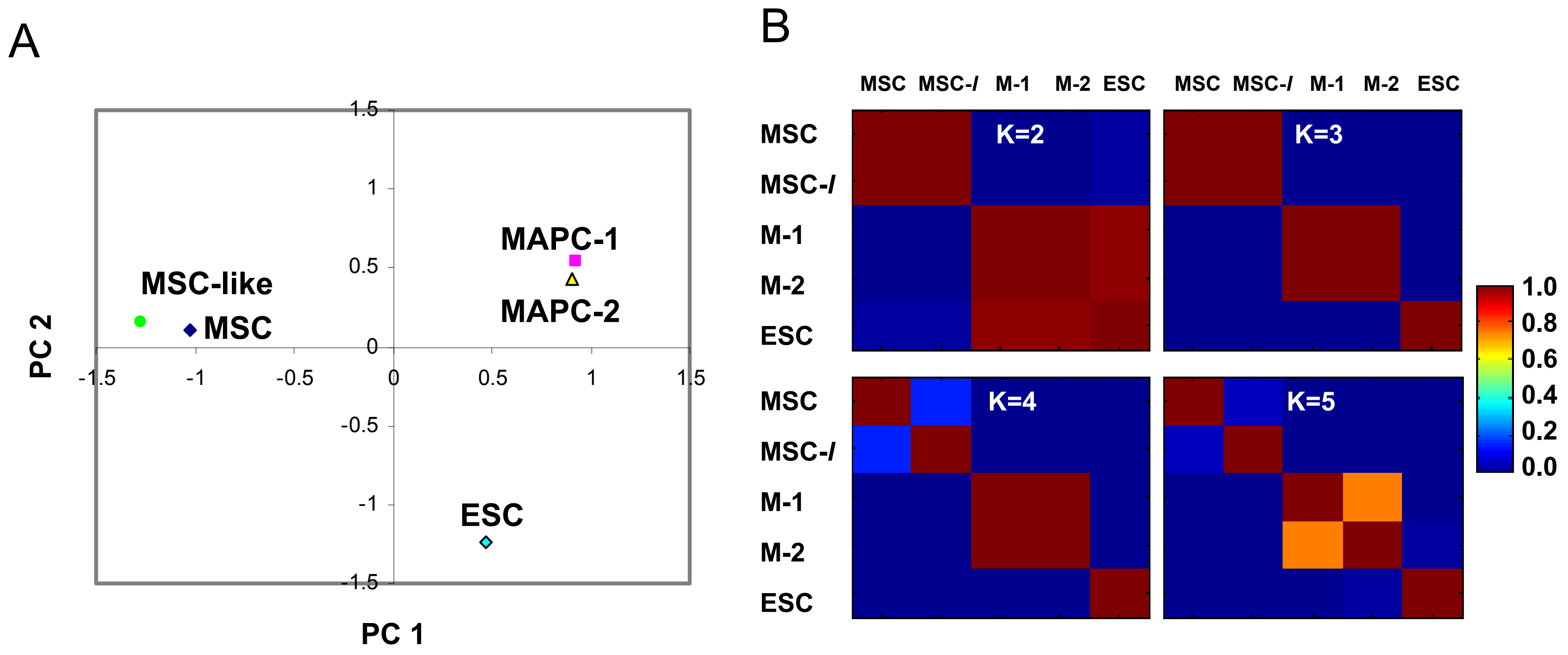

Supplementary Figure 3

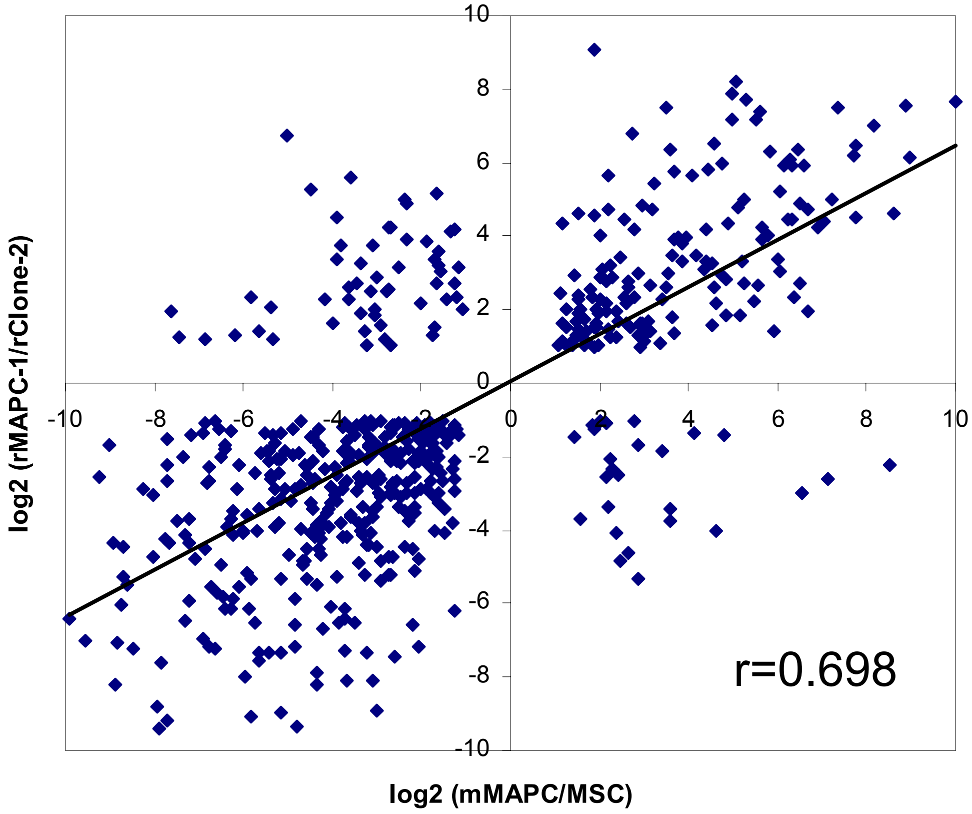

Supplement: Supplementary file 2 — Additional data file 2: Supplementary Figure 1 shows PCA of MSC, MSC-like (MSC-l, mClone-3), MAPC-1 (M-1), MAPC-2 (M-2) and ESC on reported ESC and MSC hypoxia upregulated genes; supplementary Figure 2 shows PCA and NMF analysis of MSC, MSC-like (MSC-l, mClone-3), MAPC-1 (M-1), MAPC-2 (M-2) and ESC on differentially expressed genes minus genes correlated or anticorrelated to Pdgfra; supplementary Figure 3 shows the correlation of fold difference on common differentially expressed genes between mMAPCs vs MSCs and rMAPC-1 vs rClone-2. (PDF 88 KB) [file 13059_2007_1644_MOESM2_ESM.pdf]
